# Mapping the potential of Natural Pest Control services in pan-European landscapes at 50 m resolution

**DOI:** 10.1038/s41597-025-06138-7

**Published:** 2025-11-25

**Authors:** Rui Catarino, Ana Klinnert, Ana Luisa Barbosa, Raphael d’Andrimont, Thomas Fellmann, Renate Koeble, Maria Luisa Paracchini, Jean-Michel Terres, Marijn van der Velde, Peter Vogt, Carlo Rega

**Affiliations:** 1 European Commission, Joint Research Centre (JRC), Ispra, Italy; 2 European Commission, Joint Research Centre (JRC), Seville, Spain; 3 European Commission, Directorate-General Agriculture and Rural Development, Brussels, Belgium; 4 ARHS Developments S.A., Belvaux, Luxembourg

**Keywords:** Agroecology, Ecological modelling, Ecosystem services

## Abstract

Natural pest control is a crucial regulating ecosystem service for sustaining crop yields while lowering pesticide use. Its effectiveness depends on landscape complexity, particularly the amount and spatial arrangement of semi-natural habitats, in and around fields, that support natural enemies. We provide a 50 m-resolution, pan-European map of NPC potential that updates and refines the model of Rega *et al*. (2018). The workflow merges Copernicus High-Resolution Layers for 2018 - Tree Cover Density, Grasslands and Woody Vegetation Mask (5–10 m source grids) - with empirical coefficients from extensive field surveys. Morphological spatial pattern analysis distinguishes linear from areal habitat elements, capturing their contrasting ecological functions: linear features improve connectivity for enemy movement, whereas areal patches offer stable refugia and resources. All scripts and configuration files are openly released, allowing straightforward reruns with future Copernicus updates or with analogous land-cover products outside Europe. The resulting NPC index maps spatial variation in habitat composition, abundance and proximity to cropland, providing an indicator for planning and management from continental to local scales.

## Background & Summary

The assessment and mapping of Ecosystem Services (ES) are essential for designing effective environmental management and conservation policies^1,2^. With the growing availability of high-resolution, spatially explicit data, ES mapping has become integral to data-driven policymaking, enabling national and local authorities to target interventions where supply and demand diverge, while engaging key stakeholders such as farmers, advisers, and conservation groups^3–5^, particularly within agroecosystems^6^. Such spatial evidence underpins EU strategies for sustainable and resilient agriculture, including the Nature Restoration Regulation^7^.

Among regulating services, biodiversity-driven natural pest control (NPC) plays a crucial role in reducing pesticide reliance, sustaining yields^8–10^, and enhancing farm resilience^10,11^. The potential for NPC depends strongly on landscape complexity, particularly the presence, abundance, and configuration of semi-natural habitats (SNH). These habitats support the abundance and diversity of natural enemies (NE) of crop pests and help stabilize ecological interactions across scales^12,13^. This means that ecosystem service potential, defined as the maximum capacity to deliver services irrespective of demand, is shaped by properties such as SNH extent, patch connectivity, crop diversity^3,14,15^. While increasing SNH does not always guarantee enhanced NPC^16^, it provides the ecological conditions necessary for sustaining it^17^. These same SNH types also support other services including pollination, soil fertility, carbon sequestration, and water regulation^4,18^, reinforcing the multifunctional value of such features in agricultural landscapes. Recent work has further highlighted the importance of restoring farmland biodiversity to enhance NPC^19^, and the need for robust models to predict pest control dynamics and contribution across agricultural landscapes^10,20,21^.

Rega *et al*.^22^ produced the first pan-European Natural Pest Control Index (NPCi) at 100 m resolution, combining field-derived coefficients with coarse land-cover data. Other mapping efforts^23,24^ lacked landscape configuration metrics, predator data, or detailed field validation. With the release of Copernicus HRLs (5–10 m), it is now possible to refine NPC assessment to a scale relevant for farm and field management.

Here we present an updated 50 m-resolution pan-European map of NPCi. The workflow integrates three 2018 HRLs - Tree Cover Density, Grasslands and Woody Vegetation Mask - with coefficients derived from 217 field sites that quantified flying NE^25^. A morphological spatial pattern analysis classifies SNH as linear or areal, capturing their distinct ecological functions: linear elements facilitate movement and connectivity, whereas areal patches offer stable refuges and resources. Extensive grasslands are identified using a CAPRI nitrogen-input layer (<50 kg N ha^−1^ yr^−1^) as a proxy for low-intensity management^26^.

By increasing the spatial detail from 100 m to 50 m, the updated NPCi captures fine-scale heterogeneity that drives predator dynamics, enabling analyses ranging from continental monitoring down to field-scale planning. It supports scenario modelling of SNH enhancement, exploration of synergies with other services such as pollination, and evaluation of agri-environmental measures aimed at for example reducing pesticide risk. To facilitate broader uptake, we also provide ancillary layers on SNH configuration and landscape composition, enhancing compatibility with other ES datasets and supporting integrated environmental assessments across scales. All scripts and GeoTIFF layers (EPSG:3035), released under CC-BY 4.0 and archived in the JRC Data Catalogue^27^, follow FAIR principles^28^. The workflow is modular and adaptable, allowing users to incorporate updated HRLs or apply the method in other regions using equivalent land-cover data.

## Methods

The methodology follows Rega *et al*.^22^, who used extensive field data^25^ to quantify how NE - predatory flies and parasitic wasps - respond to different SNH. These surveys quantified NE abundances across 217 SNH sites in 62 agricultural landscapes in four European countries, covering woody and herbaceous habitats with different spatial morphologies (linear vs. areal) and positions (edge vs. interior). The resulting NPCi estimates the relative potential of surrounding landscapes to support NPC within a 500 m flight radius, rather than the final service delivery (e.g. yield increase or pest suppression), which remains highly context-dependent^29^. To scale the approach at the pan-European scale, we integrated five key geospatial layers describing land cover, vegetation structure, and nitrogen input (Table 1), which together provide the baseline for assessing habitat configuration across the continent.

**Table 1:** Data layers incorporated into the model.

	Forest^1^	WVM^2^	Grasslands^3^	N layer^4^	CLC^5^
Temporal extent	2018	2017–2019	2017–2018	2017 (mean 2016–2018)	2017-2018
Spatial Resolution	10 m	5 m	10 m	1 km	100 m
Coverage	Pan-Europe	Pan-Europe	Pan-Europe	EU27 (excl. HR) and UK	Pan-Europe
Type of data	% (0–100)	Binary	Binary	Kg N/ha	44 classes
Date of publication	Sep 18, 2020	May 10, 2023	May 10, 2023	Under publication	Jun 14, 2019 (revised May 13, 2020)

This table details the various data layers used to assess NPC potential across Europe, including temporal extent, spatial resolution, geographical coverage, data type, and publication date.

^1^High Resolution Layer Tree Cover Density, https://land.copernicus.eu/pan-European/high-resolution-layers/forests/tree-cover-density/status-maps/tree-cover-density-2018?tab=metadata.

^2^High Resolution Layer Woody Vegetation Mask, https://land.copernicus.eu/pan-European/high-resolution-layers/small-woody-features/small-woody-features-2018?tab=metadata.

^3^High Resolution Layer Grassland, https://land.copernicus.eu/pan-European/high-resolution-layers/grassland/status-maps/grassland-2018?tab=metadata.

^4^Nitrogen (N) layer from the CAPRI model^26^. N use in grasslands was extracted specifically for the purpose of our study. Available at https://jeodpp.jrc.ec.europa.eu/ftp/jrc-opendata/NPCI2018/?C=S;O=A.

^5^CORINE Land Cover, https://land.copernicus.eu/pan-European/corine-land-cover/clc2018?tab=metadata.

### Morphological analysis of woody elements

To derive a comprehensive classification of woody elements, we merged two High Resolution Layers (HRLs): Tree Cover Density^30^ (TCD) and Woody Vegetation Mask^31^ (WVM). The TCD layer provides values ranging from 0% to 100% at a 10 m resolution for 2018, while the WVM layer, part of the Small Woody Features 2018 dataset portfolio^32^, captures woody features regardless of size or shape, including hedgerows, tree alignments, isolated trees, and small wooded patches. Due to their differing original resolutions, the WVM layer was resampled to 10 m. A pixel was assigned a value of one if at least 50% (i.e. two out of four) of the corresponding original pixels were classified as woody, otherwise it was assigned a value of zero.

**Fig. 1:**
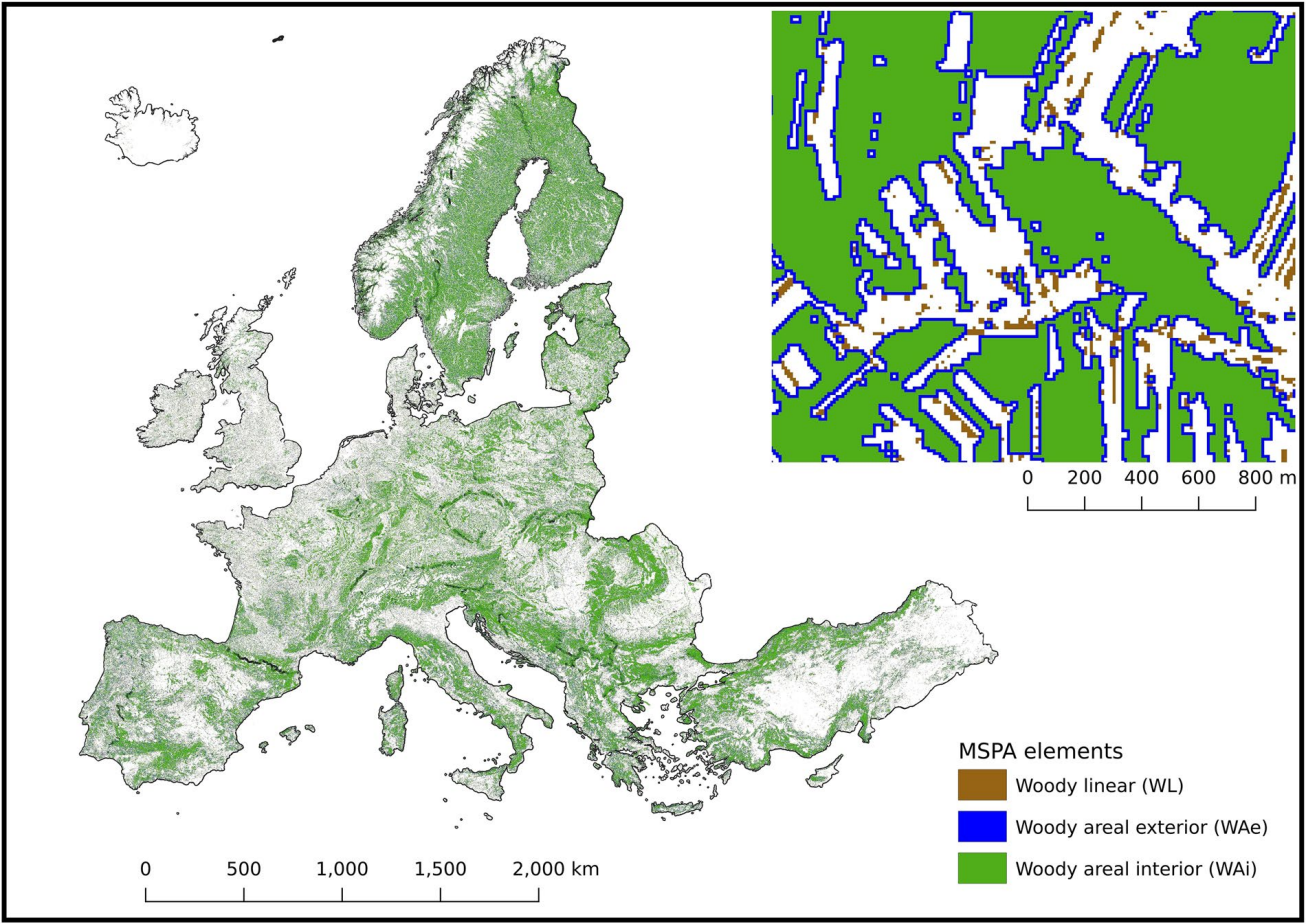
Results from morphological spatial pattern analysis (MSPA) using the open-source software GuidosToolbox Workbench (available at https://forest.jrc.ec.europa.eu/en/activities/lpa/gwb/). Woody SNH patches extending over 30 m in all directions were classified as Woody Areal (WA) elements, while any element with a width ≤30 m and a length ≥10 m was defined as Woody Linear (WL) elements. The figure shows Woody Linear (WL) in brown, representing linear woody elements; Woody Areal - Exterior (WAe) in blue, representing the 10 m boundary from the forest edge (equivalent to one pixel); and Woody Areal - Interior (WAi) in green, representing the area extending beyond the forest edge into the interior. The top-right corner displays a zoomed-in example at a scale of approximately 1:10,000, illustrating the detailed classification of MSPA elements. White areas indicate no data (non-agricultural land, outside the scope of the NPCi calculation). The corresponding TIF file can be accessed at the following link: https://jeodpp.jrc.ec.europa.eu/ftp/jrc-opendata/NPCI2018/?C=S;O=A.

Subsequently, both HRLs, now at 10 m resolution, were merged and classified by morphological spatial pattern analysis^33^ (MSPA) using the open-source software GuidosToolbox Workbench^34^ (available at https://forest.jrc.ec.europa.eu/en/activities/lpa/gwb/). MSPA classifies spatial elements based on their shape, size, and configuration, allowing differentiation between woody areal (WA) and woody linear (WL) SNH. Elements ≥30 m wide in all directions were classified as woody areal (WA); narrower elements ≥10 m long and ≤30 m wide were classified as woody linear (WL). Within WA, the edge zone (WAe) was defined as a 10 m buffer from the forest edge (one pixel), and the remainder as woody areal interior (WAi) (Fig. 1).

### Integration of extensive grasslands

The HRL for grasslands^35^ (GL) was incorporated to account for herbaceous SNH. This 10 m layer provides a pan-European binary classification (grassland/non-grassland) for the year 2018. As grassland biodiversity varies significantly with management intensity (e.g. fertilization, mowing frequency, and grazing density), only extensive grasslands were considered as supportive of rich forb communities and other herbaceous species beneficial to NEs^36^.

**Fig. 2:**
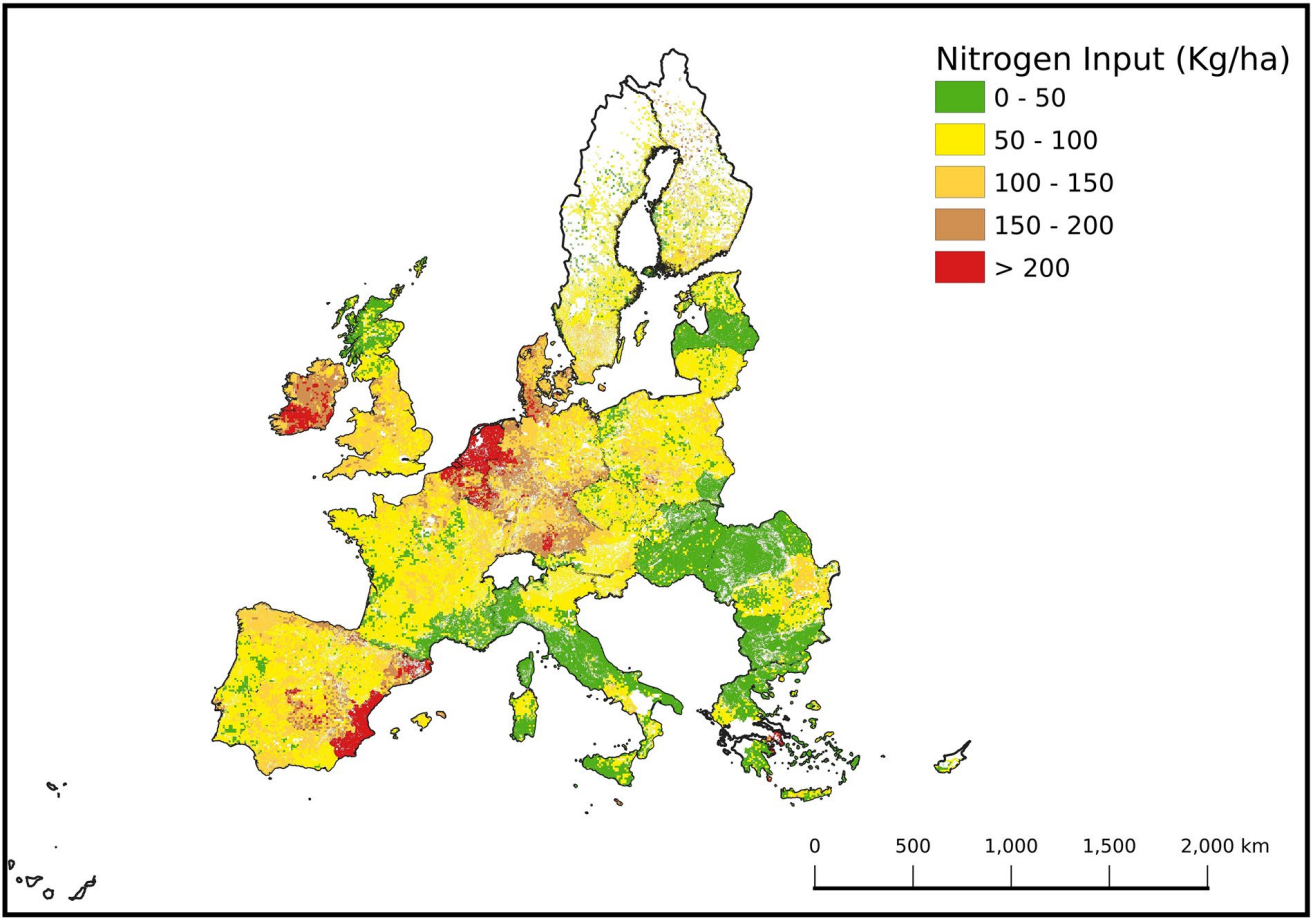
The CAPRI Nitrogen Layer was derived from the nitrogen (N) layer of the CAPRI model, mapping nitrogen input (Kg/ha) across pan-Europe with a spatial resolution of 1000 meters. It distinguishes extensive (≤50 kg/ha) from intensive grasslands in the High-Resolution Layer Grasslands 2018 (HRL GLs), serving as a proxy for low and high intensity managed grassland. The nitrogen input levels are categorized into five classes: less than or equal to 50 (green), 50 - 100 (yellow), 100 - 150 (orange), 150 - 200 (brown), and greater than 200 (red). This map highlights the spatial variability of nitrogen input in agricultural lands across different European regions. The CAPRI N layer covers the following countries: Bulgaria, Czechia, Belgium, Austria, Germany, Cyprus, Denmark, Estonia, Greece, Spain, Finland, Hungary, France, Ireland, the Netherlands, Italy, Liechtenstein, Lithuania, Luxembourg, Latvia, Malta, Portugal, Romania, Sweden, the United Kingdom, Poland, Slovenia, and Slovakia. The corresponding TIF file can be accessed at the following link: https://jeodpp.jrc.ec.europa.eu/ftp/jrc-opendata/NPCI2018/?C=S;O=A.

**Fig. 3:**
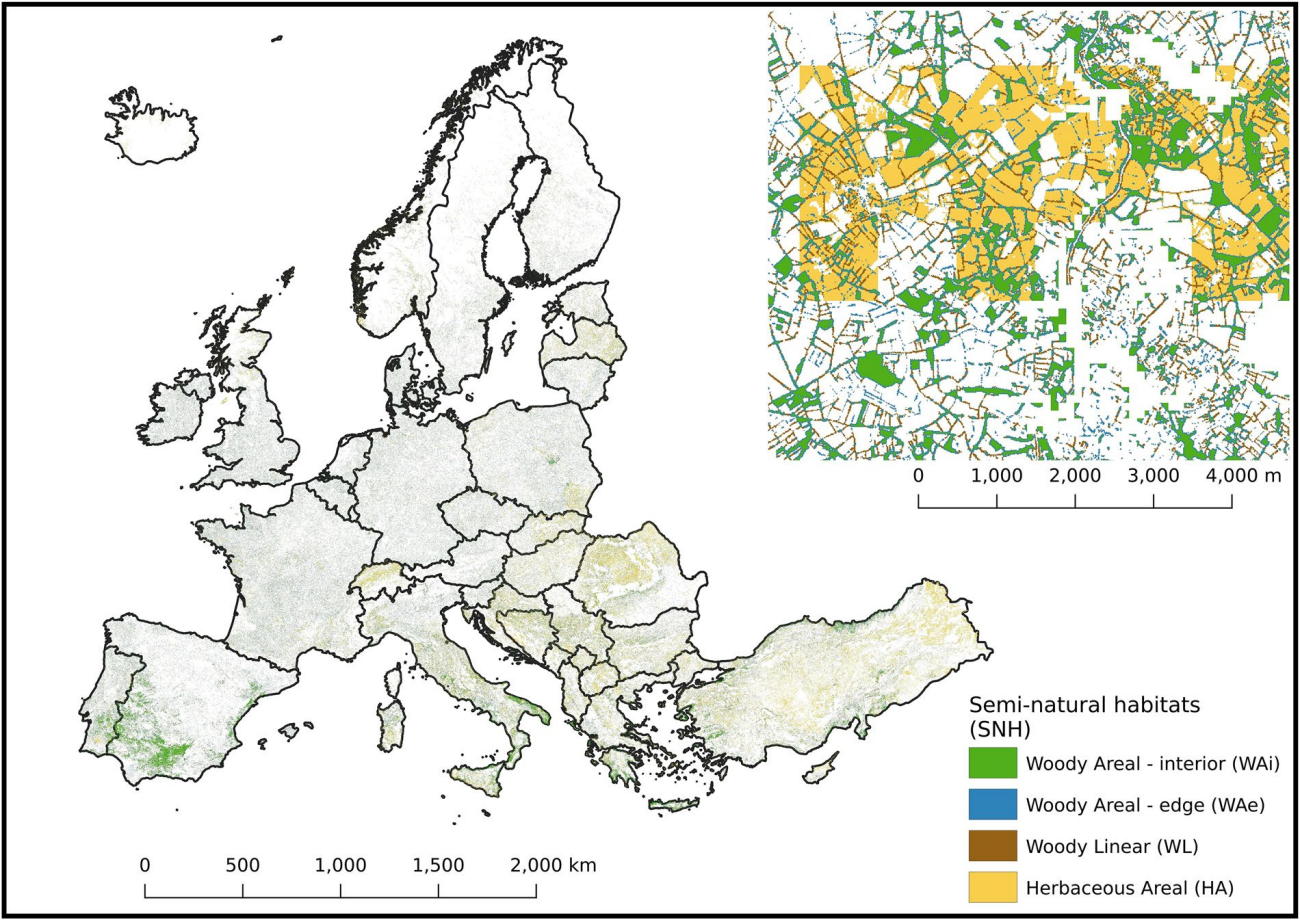
Pan-European distribution of semi-natural habitats (SNH), categorized into four types: Woody Areal - edge (WAe) (blue), Woody Areal - interior (WAi) (green), Woody Linear (WL) (brown), and Herbaceous Areal (HA) (yellow). Extensive grasslands were classified as HA and integrated with the woody elements layer, previously classified through morphological analysis. The respective areas at the pan-European level are as follows: HA covers 228,593 km², WL covers 49,786 km², WAi covers 215,384 km², and WAe covers 139,492 km². Agricultural Land covers a total of 2,423,947 km². These areas represent 9.4%, 2.1%, 8.9%, and 5.8% of the total agricultural area, respectively, with Agricultural Areas constituting 40.9% of the total land area. The top-right corner displays a zoomed-in example at a scale of approximately 1:4,000, illustrating the detailed classification of SNH elements. The corresponding TIF file can be accessed at the following link: https://jeodpp.jrc.ec.europa.eu/ftp/jrc-opendata/NPCI2018/?C = S;O = A.

Since the HRL-GL does not differentiate intensity levels, along with the lack of such data at the pan-European level, we used the nitrogen (N) input layer^26^ from the CAPRI model^37,38^ as a proxy (Fig. 2). This dataset disaggregates nitrogen inputs to crops and grasslands across the EU27 (excluding Croatia) and the UK. Grasslands receiving <50 kg N ha^−1^ yr^−1^ were classified as extensive^39^. In countries not covered by this layer, we assumed all grasslands to be extensive. These selected areas were then classified as Herbaceous Areal (HA) and merged with the previously classified woody SNH layer (Fig. 3).

### Final pan-european level NPCi layer

The SNH are classified into four types (Table 2) based on the predominant vegetation type - woody or herbaceous - and their morphological shape: Woody Areal edge (WAe), Woody Areal interior (WAi), Woody Linear (WL), and Herbaceous Areal (HA). Their estimated potential contributions to NPC were based on average NE abundances^25^ for each SNH type and location.

**Table 2:** Potential NPC scores across different types of SNH in Europe.

SNH type	Score
Herbaceous Areal (HA)	26.8
Woody Areal – edge (WAe)	45.6
Woody Areal – interior (WAi)	20.7
Woody Linear (WL)	34.4

The scores represent the average abundances of flying predators predicted by the model. Note that these scores do not have absolute significance but are used to assess relative potential across different SNH types and within-SNH locations.

Source^22,25^:

The classified 10 m SNH layer was resampled to 50 m resolution using nearest neighbour to reduce computational load and preserve the categorical structure^22^, avoiding the introduction of mixed or interpolated values that could distort the classification. The potential NPCi for each 50 m cell was calculated by summing the contribution of all SNH elements within a 500 m radius, weighted by a distance-decay function. This approach is consistent with other spatially explicit ecosystem service models^2–5,40–42^.

To isolate agricultural land, the CORINE Land Cover^43^ (CLC) dataset was used to mask out non-agricultural areas. The CLC provides a pan-European inventory for 44 thematic classes for the 2018 reference year^44^, with a resolution of 100 meters. We retained four selected CLC agricultural classes (Supplementary Table 1):

- Arable land: Areas used for annually harvested crops, including flooded crops (e.g., rice) and fallow lands.
- Permanent crops: Areas dedicated to non-rotational crops, such as orchards, olive groves, and vineyards.
- Pastures: Lands used permanently (at least 5 years) for fodder production, including both natural and sown meadows.
- Heterogeneous agricultural areas: Areas with mixed crops, including annual crops with permanent crops, and landscapes where crops and pastures are interspersed with natural vegetation.

**Fig. 4:**
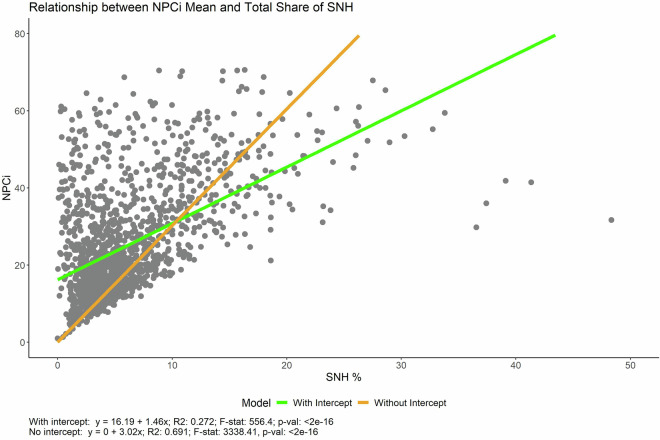
Relationship between the Natural Pest Control index (NPCi) and the Semi-Natural Habitat (SNH) share across Europe. The scatter plot shows the linear relationship between NPCi and SNH share across Europe, fitted with two linear models: one with an intercept (in green) and one forced through the origin (in orange). For the model with an intercept, the equation is y = 16.19 + 1.46x, with an R² value of 0.272, an F-statistic of 556.4, and a p-value of <2e-16. For the model forced through the origin, the equation is y = 0 + 3.02x, with an R² value of 0.691, an F-statistic of 3338.41, and a p-value of < 2e-16. The forced-origin model assumes that no semi-natural habitat (SNH) corresponds to no natural pest control (NPCi), aligning with the theoretical expectation that natural habitats are necessary for pest control. This biologically meaningful approach simplifies the interpretation of results and reflects the dependency of natural pest control on the availability of semi-natural habitats.

Although the model architecture inherently links NPCi estimates to SNH share, our pan-European results reveal important variation in NPCi values among regions with similar SNH proportions (Supplementary Table 2; Fig. 4). This confirms that SNH configuration, not just extent, strongly influences NPC potential. Consequently, agri-environmental measures and spatial planning strategies can be more effective when focusing on optimising the layout and connectivity of SNH rather than merely increasing area indiscriminately.

Six countries (Cyprus, Iceland, Liechtenstein, Luxembourg, Malta and Montenegro) showed non-significant results with low R² values or insufficient degrees of freedom (Supplementary Table 2). These countries were excluded in the subsequent descriptions provided due to lack of statistical robustness.

## Data Records

The full dataset is available at the JRC data catalogue platform^27^. The geospatial raster files generated through this processing pipeline form the core dataset for this study. These high-resolution files provide critical inputs for analysing the potential of landscapes to support NPC services. Each file serves a specific purpose within the modelling framework, as described below:

- NFIELD_2017.tif: Represents nitrogen field levels for 2017, derived from the CAPRI model. This raster provides spatially explicit data on nitrogen availability, essential for understanding its impact on vegetation and pest control dynamics.
- NPCi_2018.tif: Contains the NPCi for 2018, a key ecosystem service indicator derived from landscape composition and complexity, highlighting areas with high and low pest control potential.
- SNH_2018.tif: Maps SNH across Europe for 2018, categorizing them into four types: Woody Areal - Edge (WAe), Woody Areal - Interior (WAi), Woody Linear (WL), and Herbaceous Areal (HA). This file supports analyses of habitat-driven ES.
- TCD10 + WVM2018_MSPA.tif: Combines Tree Cover Density (TCD) and Woody Vegetation Mask (WVM) data, processed through Morphological Spatial Pattern Analysis (MSPA) to identify spatial configurations like edges and corridors.
- extGL_2018_010m_eu_v1_comp.tif: Provides detailed mapping of grasslands at a 10-meter resolution for 2018, supporting assessments of their contributions to biodiversity and ES.

The raster files and the processing pipeline scripts are available for download at: https://jeodpp.jrc.ec.europa.eu/ftp/jrc-opendata/NPCI2018/?C=S;O=A. These data are provided under the European Commission’s Reuse and Copyright Notice, which allows reuse with proper attribution. Users should refer to the specific dataset page on the JRC Data Catalogue for any additional terms and conditions (https://data.jrc.ec.europa.eu/dataset/963ed44c-b38f-4e9a-94db-990d5d0c93c8).

### Data overview

This section provides a descriptive overview of how differences in landscape composition and structure are reflected in the spatial distribution of NPCi values. At the European scale, the NPCi map highlights areas with relatively higher or lower potential to support beneficial flying predators.

### Semi-natural habitat data description

**Fig. 5:**
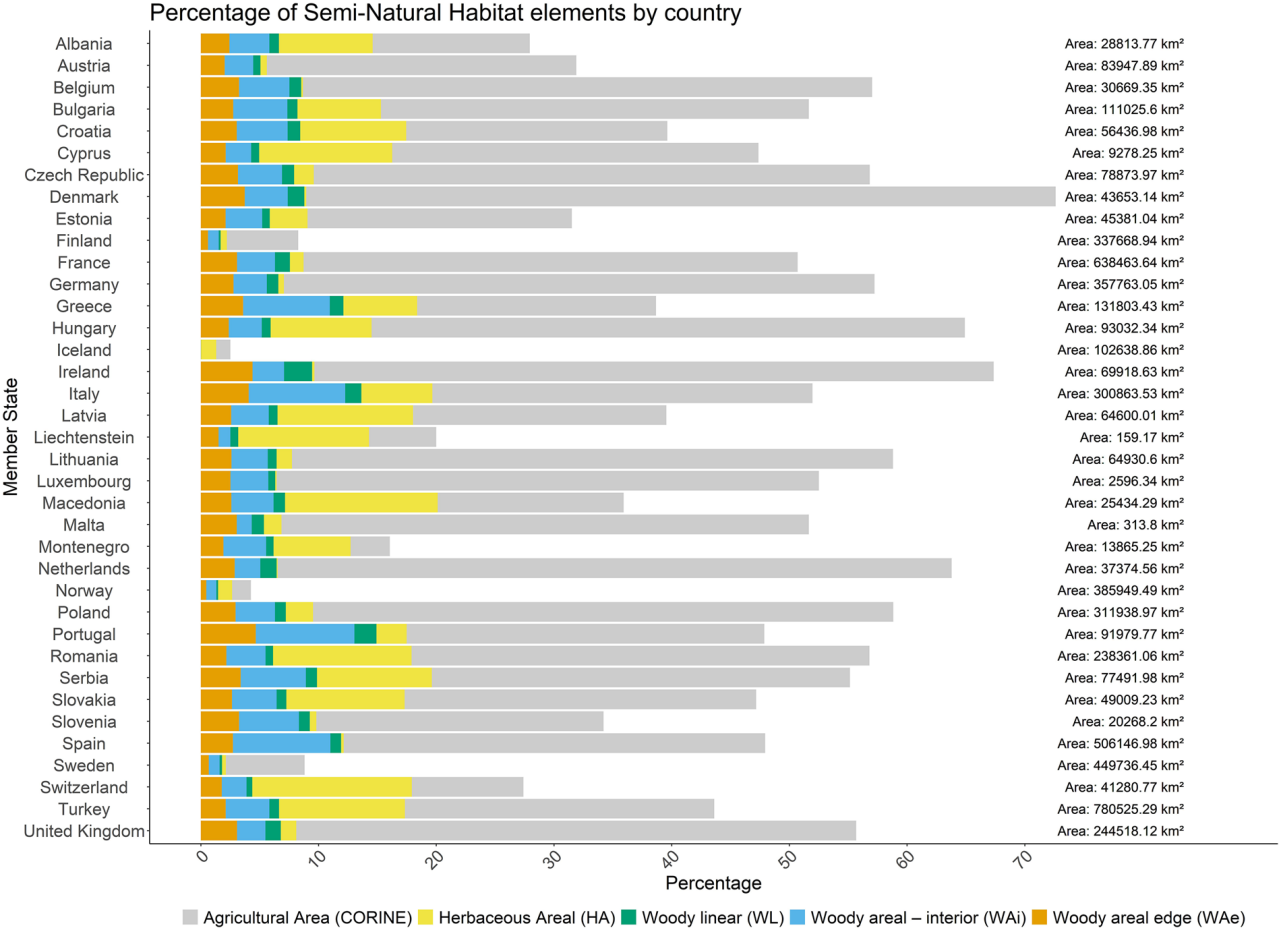
Distribution of Semi-Natural Habitat (SNH) elements and agricultural area by country across Europe. Each bar in the plot represents the different shares within each country: Herbaceous Areal (HA), Woody Linear (WL), Woody Areal - Interior (WAi), Woody Areal - Edge (WAe), and Agricultural Area (CORINE). The total area for each country is annotated in square kilometres at the far right of each horizontal bar. The total average share (±SD) of Semi-Natural Habitat elements across all countries are as follows: Agricultural Land (AgLand) 42.82 ± 18.33%; Herbaceous Areal (HA) 4.73 ± 4.65%; Woody Linear (WL) 0.89 ± 0.46%; Woody Areal - Interior (WAi) 3.48 ± 2.02%; Woody Areal - Edge (WAe) 2.57 ± 1.02%.

The SNH layer used in this study (Fig. 3) was generated by integrating two 10 m resolution datasets: the MSPA layer, based on woody vegetation, and the extensive GL layer derived according to nitrogen input thresholds. Across all national territories, the median shares of each SNH type within agricultural land are as follows: HA 2.32% (mean ± SD: 4.62 ± 4.65%), WAe 2.75% (2.71 ± 0.97%), WAi 3.36% (3.78 ± 1.99%), and WL 0.92% (0.95 ± 0.46%) (Fig. 5, Supplementary Table 3 and Fig. 4). Agricultural land overall represents a median of 47.9% (44.97 ± 17.23%) of total land area. However, national SNH profiles vary substantially, underscoring the diversity of European agricultural landscapes and the need for context-specific land management strategies.

For example, Denmark has over 31,000 km^2^ of agricultural land, covering 72.6% of its territory, but only 0.2% is classified as HA. In contrast, Hungary, with a comparable agricultural share (64.9% of 93,000 km^2^), has 8.6% HA. Other SNH types also vary: Denmark has 1.4% WL, 3.6% WAi, and 3.7% WAe, whereas Hungary has 0.8%, 2.8%, and 2.3% respectively.

Turkey has the largest absolute SNH area (~135,296 km^2^), covering 39.7% of its agricultural land and 17.3% of its national territory. Spain follows with 61,420 km² (25.3% of agricultural land). Switzerland shows the highest relative SNH share - 65.4% of agricultural land (~7,000 km^2^) - while the Netherlands has the lowest (10.2%, ~2,400 km^2^). These disparities highlight significant potential for increasing SNH shares, particularly in countries with lower relative proportions.

For WAe and WAi combined, Norway leads with 31.1% of its agricultural land (~5,100 km²), followed by Greece (28.3%) and Portugal (27.3%). In WL share, Portugal (3.9%) and Ireland (3.5%) rank highest, while France and Turkey have the largest absolute WL extents (~8,143 km² and ~6,363 km²). Switzerland has the highest HA proportion (49.5%, ~5,595 km²), but Turkey again leads in absolute HA area (~83,426 km², 24.5%). These national averages may mask considerable regional variation, which is explored in the following subsections.

### The pan-european NPCi map

**Fig. 6:**
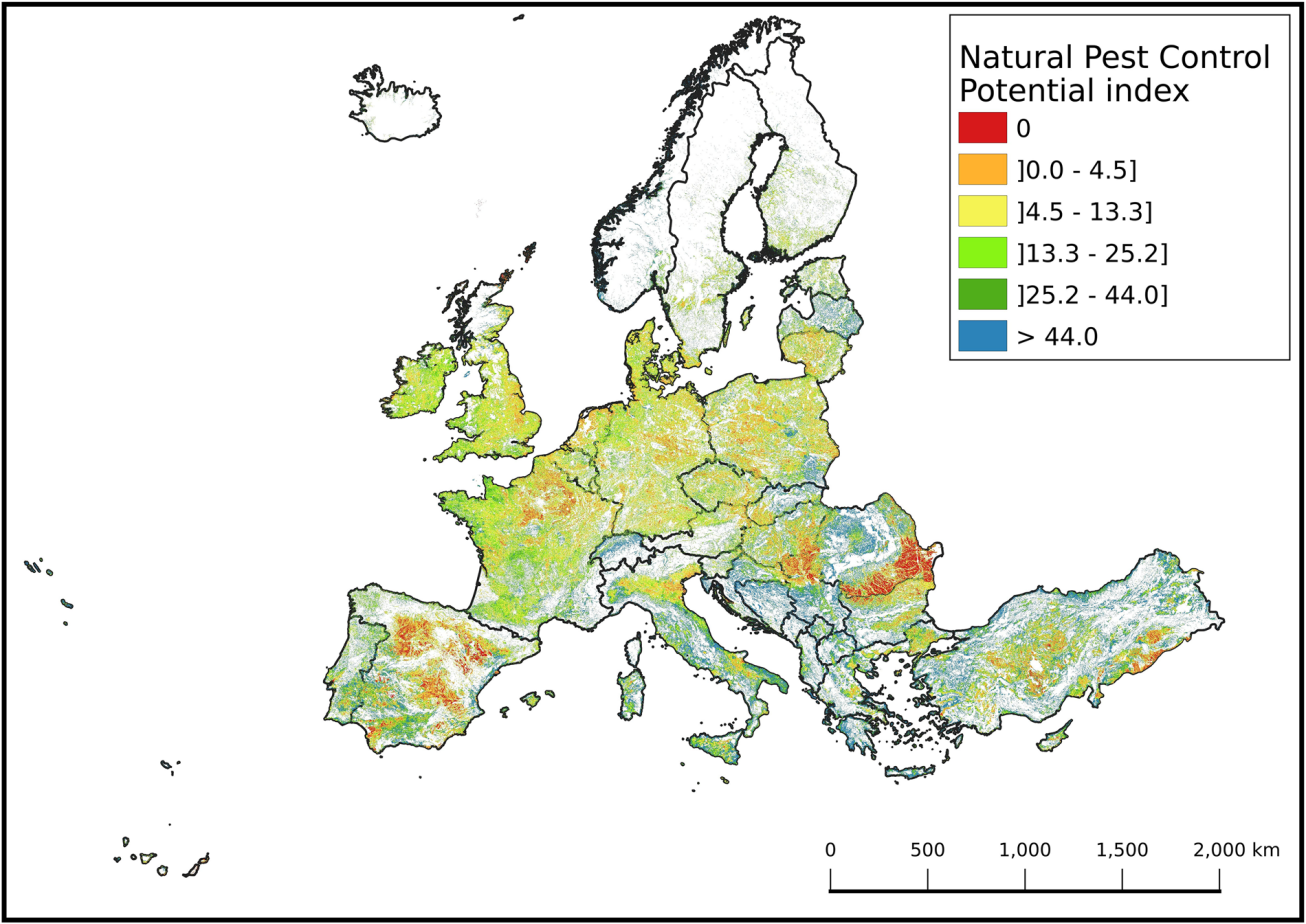
The Natural Pest Control Potential Index (NPCi) map displays the spatial distribution of NPC potential across Europe. The NPCi is a dimensionless relative score index, with values scaled from 0 to 100. Higher values (blue cells) represent cells with greater potential to support pest control, while lower values indicate lower potential (orange cells). The NPCi values are classified into quintiles based on the score distribution: equal to 0.0 (Red), 0.0 - 4.5 (Orange), 4.5 - 13.2 (Yellow), 13.2 - 25.2 (Light Green), 25.2 - 44.0 (Green), and greater than 44.0 (Blue). White areas indicate no data (non-agricultural land, outside the scope of the NPCi calculation). Spatial resolution is 50 m. The corresponding TIF file can be accessed at the following link: https://jeodpp.jrc.ec.europa.eu/ftp/jrc-opendata/NPCI2018/?C = S;O = A.

The pan-European final NPCi map (Fig. 6) shows normalized values from 0 (lowest) to 100 (highest potential) at 50 m resolution. The map reflects spatial heterogeneity in pest control potential and can guide targeted SNH restoration or enhancement. It allows practitioners to compare similar agro-climatic regions and identify low-scoring areas where SNH improvements could yield the greatest benefits.

### Characterization of NPCi at EU level

**Fig. 7:**
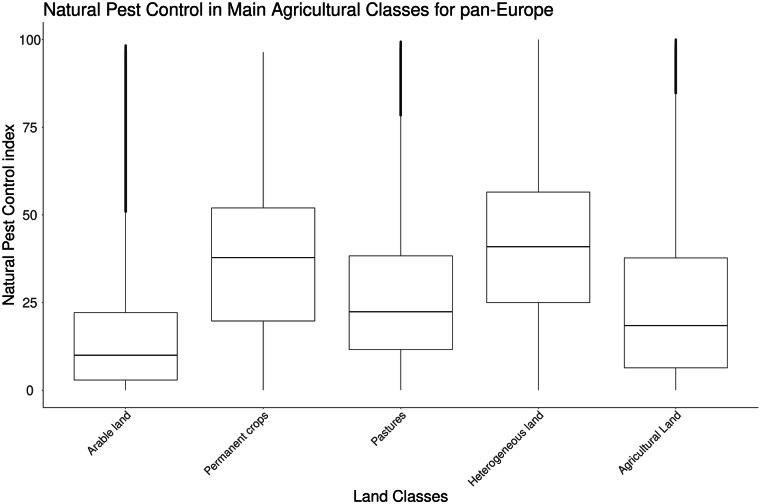
The agricultural classes include Arable Land (median 10.0, mean 15.2, SD 16.2), Permanent Crops (median 37.8, mean 35.9, SD 20.0), Pastures (median 22.4, mean 27.8, SD 21.4), Heterogeneous Land (median 47.9, mean 40.7, SD 20.3), and Agricultural Land (median 18.4, mean 24.2, SD 21.2). The NPCi values are shown on the y-axis, with the index ranging from 0 to 100. The box plots illustrate the median, interquartile range, and potential outliers for each land class, demonstrating the variability in pan-European NPC potential across different agricultural landscapes.

The NPCi reveals substantial spatial variation across Europe (Fig. 7). The mean NPCi value across agricultural areas is 24.5 ( ± 21.4), with a median of 18.7. Lower percentiles (10th: 1.3; 25th: 6.5) suggest that many agricultural areas have limited NPC potential. Analysing the NPCi by main agricultural classes provides further insights into this spatial variability.

As shown in Fig. 7, at the pan-European scale, arable land shows the lowest NPCi scores (median: 10.0; mean: 15.2 ± 16.2), pastures follow (median: 22.4; mean: 27.8 ± 21.4), permanent crops score higher (median: 37.8; mean: 35.9 ± 20.0) and heterogeneous agricultural areas rank highest (median: 40.9; mean: 40.7 ± 20.3). These patterns reflect differences in land use intensity and structural complexity, which influence habitat heterogeneity and SNH presence. Arable land, dominated by monocultures, offers few ecological niches. In contrast, permanent crops and heterogeneous mosaics incorporate SNH elements, particularly WAe and WL, that promote connectivity and predator movement, increasing overall NPC potential.

### Comparison of NPCi across European countries

NPCi values differ widely across countries (Supplementary Figure 1a–d, Table 3). Switzerland, Norway, and Albania have the highest median NPCi values, with Switzerland showing a median of 59.3 (mean 55.2 ± 19.4), Norway with a median of 53.6 (mean 50.9 ± 17.5), and Albania with a median of 48.6 (mean 45.7 ± 19.2). High NPCi values in these countries align with large areas of SNH, particularly WAe and WAi elements.

At the lower end, the Netherlands, Denmark, and Lithuania show medians of 8.9 (mean 11.7 ± 10.6), 11.1(mean 13.8 ± 10.6), and 11.3 (mean 15.2 ± 14.1). Large agricultural producers like Germany (median: 12.1, mean 14.7 ± 12.1), Spain (median 12.7, mean 19.2 ± 19.1), and France (median 16.1, mean 18.9 ± 15.0) exhibit modest scores due to intensive practices and limited SNH. For instance, SNH covers only 12.2% of Germany’s and 17.3% of France’s agricultural land. Spain fares slightly better (25.3%), but the spatial arrangement of SNH appears suboptimal, underlining the importance of not just SNH area, but its configuration within the landscape.

## Technical Validation

Enhancing NPC services across Europe requires pinpointing regions with varying capacities to support beneficial flying predators. Through a descriptive interpretation, we verify and illustrate the internal consistency of the dataset by focusing on distinct NUTS-3 regions with differing capacities to support natural enemies (see Supplementary Table 4 for details).

**Fig. 8:**
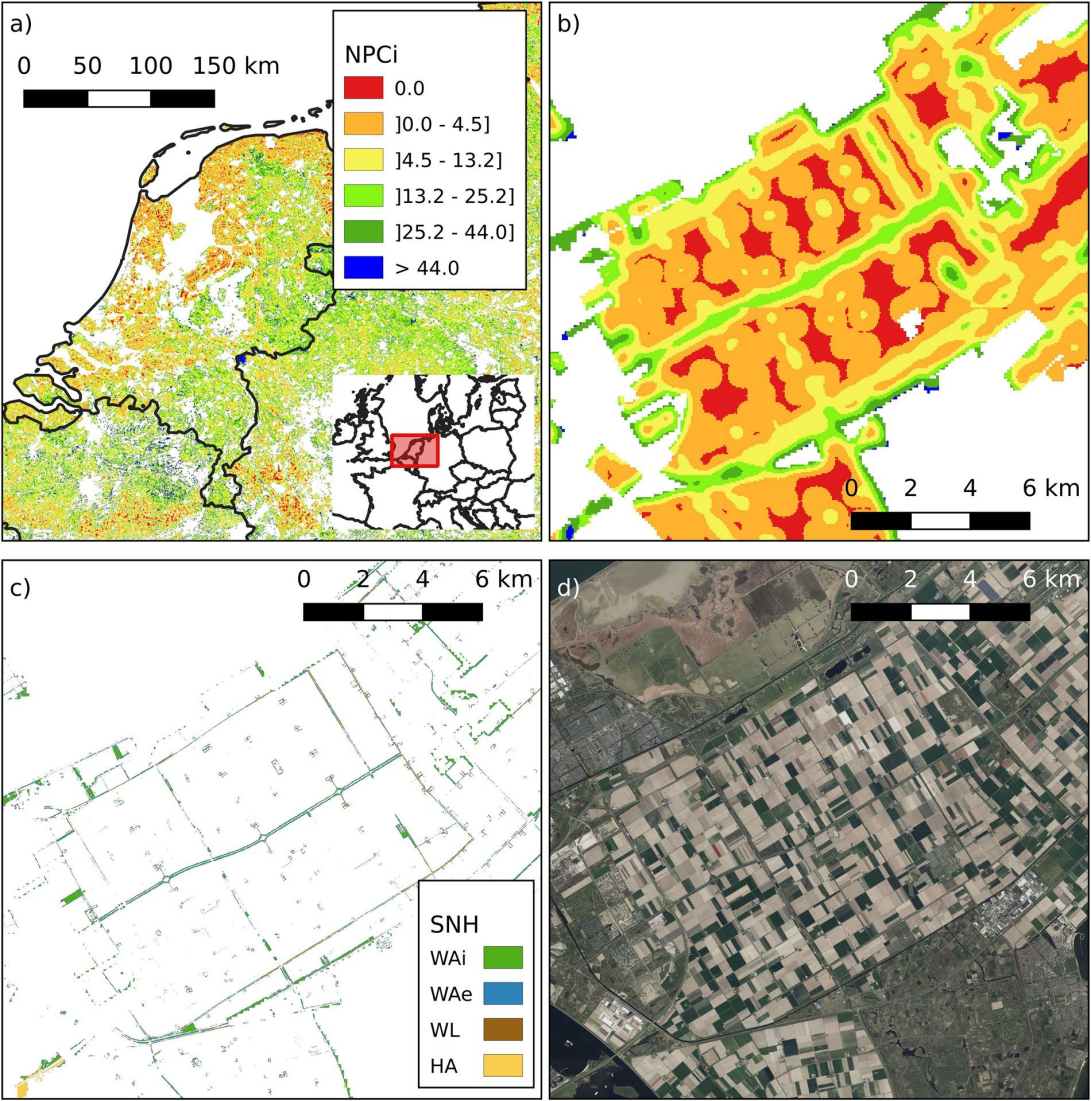
Spatial analysis of NPCi and SNH in Flevoland, Netherlands (NUTS 3: NL230). Panel a) shows the NPCi for the Netherlands, with the highlighted square indicating the location of Flevoland. Panel b) is a zoomed-in view of the NPCi within a small area in Flevoland, demonstrating the distribution of natural pest control potential. Flevoland shows a median NPCi of 1.1 and a mean of 6.0 ± 7.5. Panel c) shows, for the same location in panel b), the SNH elements, illustrating the scarcity of SNH elements in the region. The SNH categories include Woody Linear (WL), Woody Areal - Exterior (WAe), Woody Areal - Interior (WAi), and Herbaceous Area (HA). The SNH proportion in agricultural land for this region is as follows: WAe 2.1%, WAi 1.3%, WL 1.1%, and HA 0.1%. Panel d) is a satellite image of the landscape from Google Earth. White areas indicate no data (non-agricultural land, outside the scope of the NPCi calculation).

**Fig. 9:**
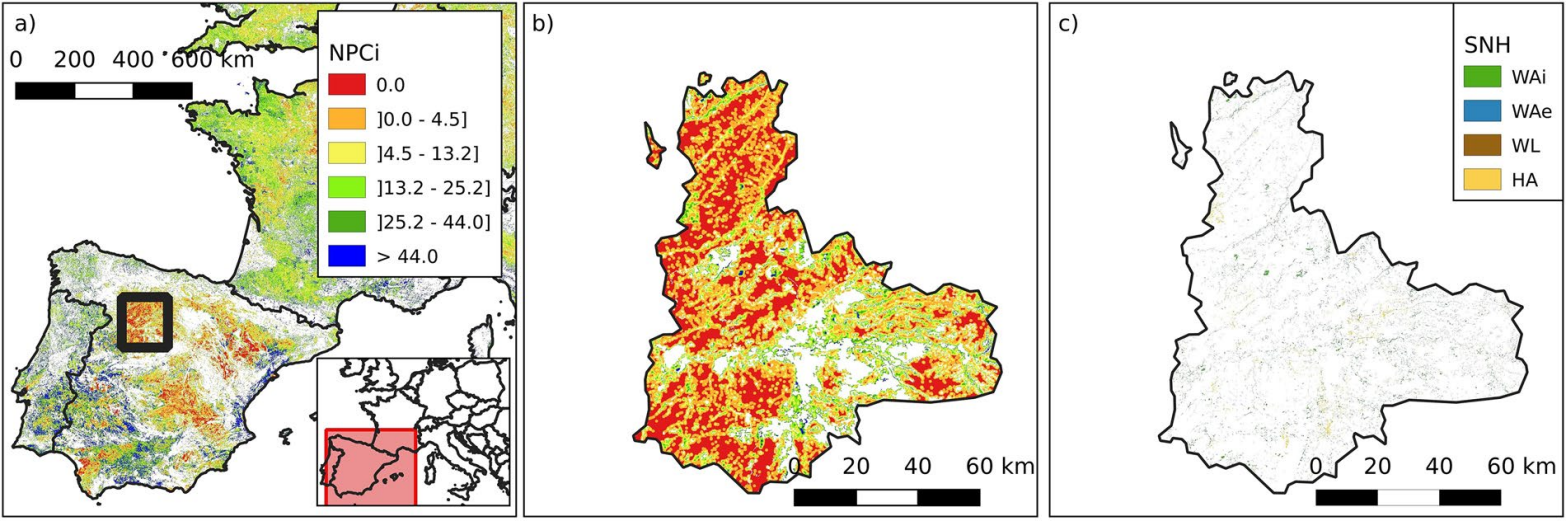
Spatial analysis of NPCi and SNH in Valladolid, Castile and León, Spain (NUTS 3: ES418). Panel A (left, scale = 50 km) shows the NPCi for Spain, with the highlighted square indicating the location of Valladolid. Panel B (middle, scale = 50 km) is a zoomed-in view of the NPCi within the region of Valladolid, demonstrating the distribution of natural pest control potential. Valladolid shows a median NPCi of 0.14 and a mean of 4.38 ± 8.22. Panel C (right, scale = 10 km) presents the SNH layer for Valladolid, showing the distribution and scarcity of SNH elements in the region. The SNH categories include Woody Linear (WL), Woody Areal - Exterior (WAe), Woody Areal - Interior (WAi), and Herbaceous Area (HA). The SNH proportion in agricultural land for this region is as follows: WAe 0.78%, WAi 0.99%, WL 1.06%, HA 0.75%. White areas indicate no data (non-agricultural land, outside the scope of the NPCi calculation).

Two regions with some of the lowest NPCi values in Europe are Valladolid (NUTS 3: ES418) in north-western Spain and Flevoland (NUTS 3: NL230) in the central Netherlands, with median scores of 1.1 (mean 5.2 ± 8.5) and 3.2 (mean 6.0 ± 7.5), respectively. Flevoland (Fig. 8), a region reclaimed from the sea, represents one of Europe’s most intensively managed agricultural landscapes. Its flat, homogenous terrain and mild Atlantic climate support high-yield production of potatoes, sugar beets, cereals, and vegetables, but the landscape is largely devoid of semi-natural elements. Similarly, Valladolid (Fig. 9), located in the Duero basin and characterized by a continental Mediterranean climate, is dominated by extensive cereal cultivation in large, uniform plots, primarily wheat and barley. In both regions, long-standing emphasis on production efficiency has led to simplified land-use patterns and low structural heterogeneity. The absence of woody features, limited edge habitats, and scarcity of extensively managed grasslands constrain the availability of ecological niches for NE. As a result, these regions exhibit minimal potential for landscape-driven NPC.

**Fig. 10:**
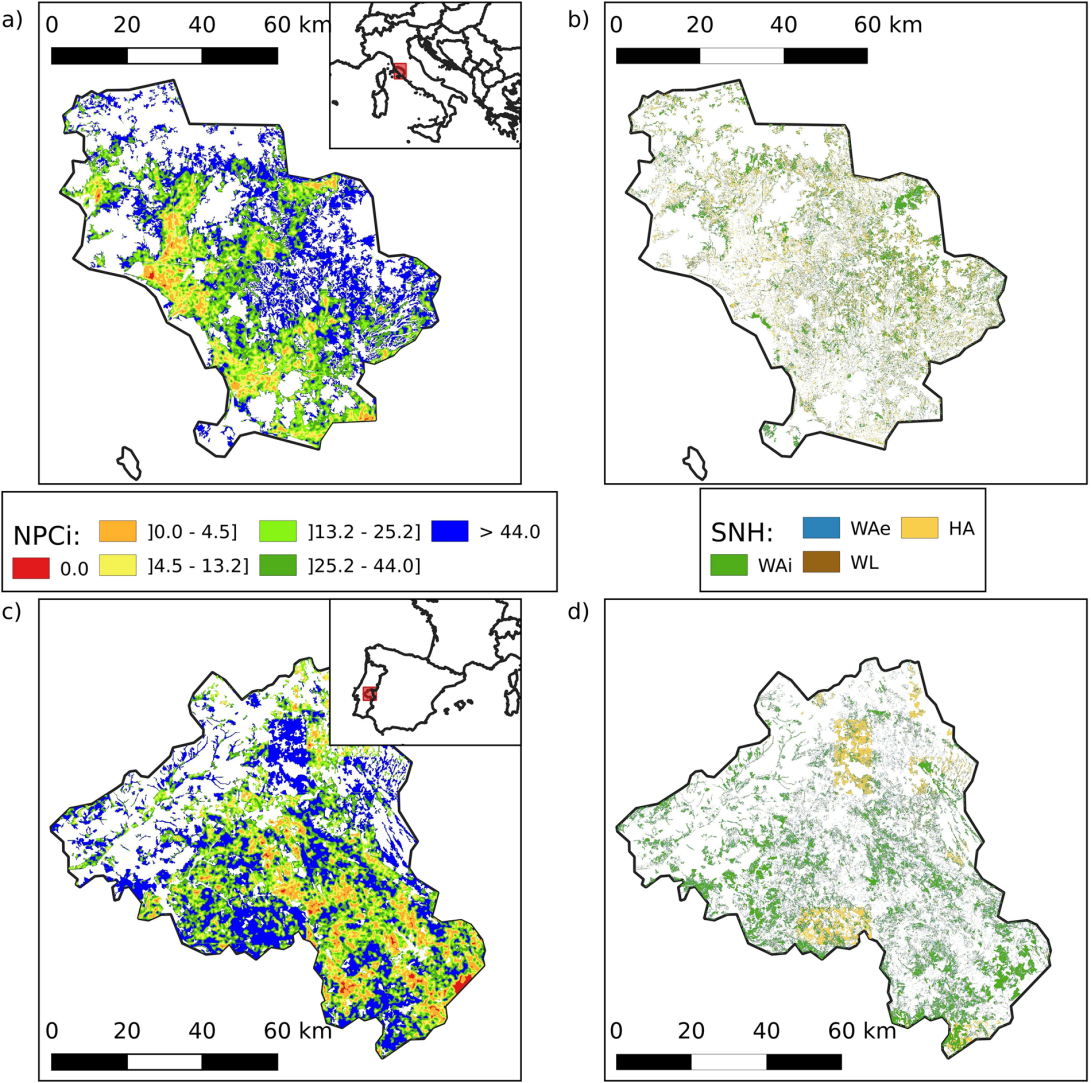
Comparative analysis of the high values for the Natural Pest Control potential Index (NPCi, left panels) and Semi-Natural Habitats (SNH, right panels) in Grosseto (NUTS 3: ITI1A), Italy (top) and Alto Alentejo (NUTS 3: PT186), Portugal (bottom). The left panels (a and c) illustrate NPCi values, with higher values indicating greater potential for natural pest control. The right panels (b and d) show the distribution of SNH, including Woody Areal – edge (WAe, light blue), Woody Areal – interior (WAi, blue), Woody Linear (WL, brown), and Herbaceous Areal (HA, yellow). The NPCi index in Grosseto and Alto Alentejo is respectively 35.4 (36.6 ± 20.9) and 32.9 (33.9 ± 21.5). Grosseto’s SNH is composed of 8.5% WAe, 12.1% WAi, 3.7% WL, and 15.4% HA. Alto Alentejo’s SNH is composed of 10.4% WAe, 17.9% WAi, 4.3% WL, and 5.0% HA. White areas indicate no data (non-agricultural land, outside the scope of the NPCi calculation).

Conversely, two regions known for their productive arable farming - Grosseto in Tuscany, central Italy (NUTS 3: ITI1A, median NPCi: 35.4; mean: 36.6 ± 20.9) and Alto Alentejo in southern Portugal (NUTS 3: PT186, median NPCi: 32.9; mean: 33.9 ± 21.5) - display relatively high NPCi values (Fig. 10). Both areas lie within Mediterranean climate zones, although Alto Alentejo experiences more marked seasonal extremes. These conditions support the cultivation of olives, grapes, wheat, and sunflowers. Importantly, the agricultural landscapes in both regions are highly fragmented, comprising a mix of large and small fields embedded within a matrix of natural or semi-natural elements. Land uses such as olive groves, vineyards, agroforestry systems, and cereal plots contribute to a structurally diverse mosaic. This landscape heterogeneity fosters ecological balance by enhancing habitat availability and connectivity for NE. Notably, Grosseto achieves high NPCi scores despite having almost no grasslands, highlighting the critical role of woody features in supporting NPC.

**Fig. 11:**
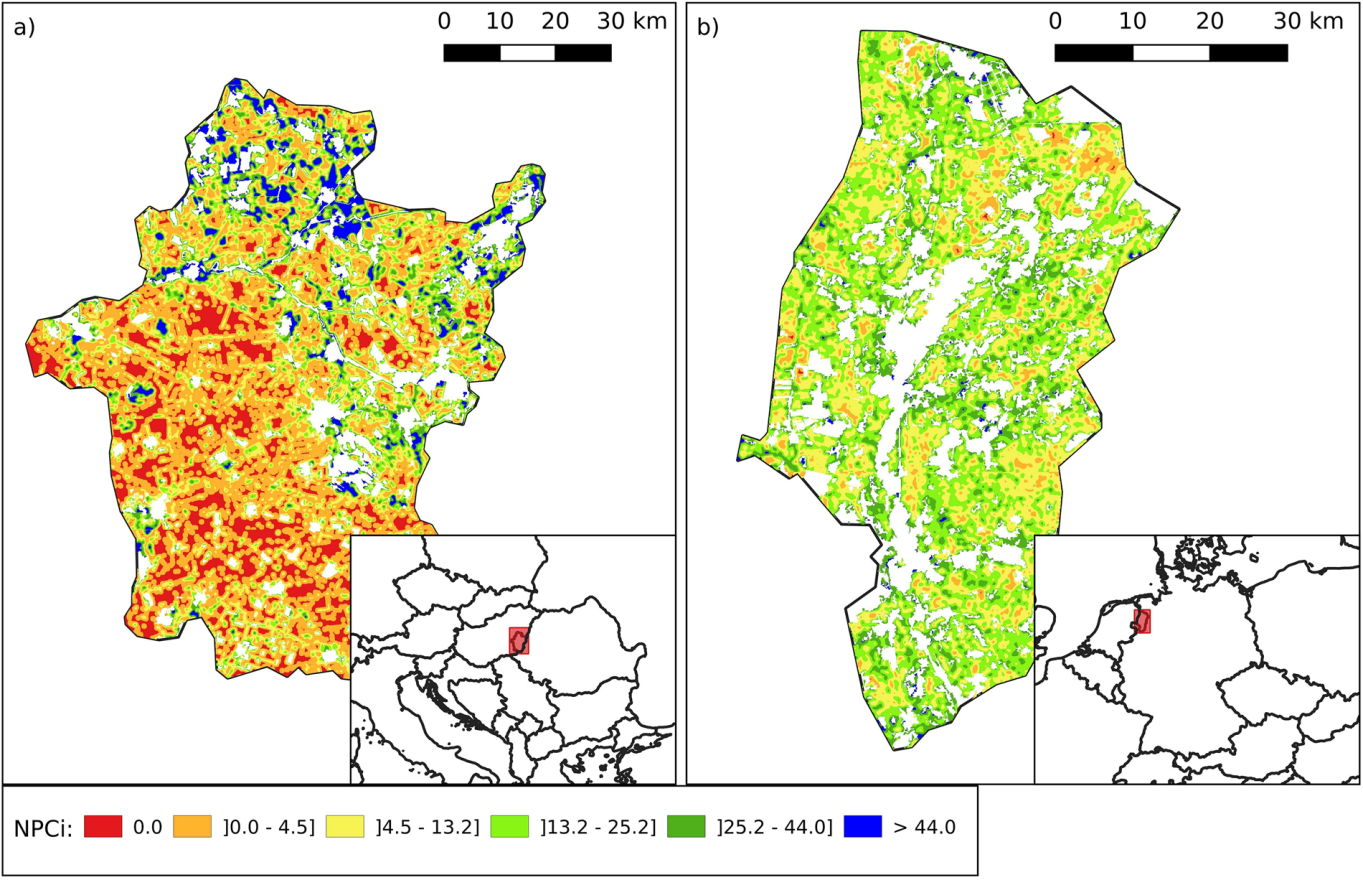
Spatial distribution of contrasting values for the Natural Pest Control index (NPCi) in two European regions: Szabolcs-Szatmár-Bereg in Hungary (NUTS3: HU332) on the left (panel a) and Emsland in northern Germany (NUTS3: DE949) on the right (panel b). The maps illustrate varying capacities to support natural pest control services, with colour gradients representing NPCi values. Szabolcs-Szatmár-Bereg (left) has 90.5% of its land dedicated to arable farming and exhibits a low NPCi with a median value of 3.5 and a mean of 9.7 ± 14.6. Emsland (right), located in the region of Lower Saxony, Germany, has 90.2% of its land dedicated to arable farming and shows a higher NPCi with a median value of 15.1 and a mean of 16.6 ± 9.2. The SNH proportions in Szabolcs-Szatmár-Bereg are WAi 1.4%, WAe 1.7%, WL 0.6%, and HA 5.8%, while in Emsland they are WAi 4.9%, WAe 5.8%, WL 3.0%, and HA 0.4%. These maps provide a visual comparison of the potential for natural pest control in regions with similar proportions of arable land but differing NPCi values. White areas indicate no data (non-agricultural land, outside the scope of the NPCi calculation).

As previously discussed, regions dominated by arable land tend to exhibit lower NPCi values due to the limited presence of semi-natural features. Hungary (NPCi median: 15.0; mean: 21.3 ± 20.3), where 78.1% of agricultural land is arable, follows Denmark (median: 11.1; mean: 13.8 ± 10.6), which has the highest arable share in Europe at 83.8%. Within Hungary, the region of Békés (NUTS3: HU332; Fig. 11a), situated in the fertile Pannonian Plain, stands out with 90.5% of its land dedicated to arable farming. Historically shaped by collectivization during the Soviet era, Békés underwent large-scale farm consolidation, reducing landscape heterogeneity. Although land ownership has since shifted to smaller private holdings, intensive farming systems persist, leaving little room for structural elements such as hedgerows or uncultivated margins. These landscape simplifications contribute to its low NPCi (median: 3.5; mean: 9.7 ± 14.6), despite the region’s agricultural prominence.

In comparison, Emsland (NUTS3: DE949; Fig. 11b) in northern Germany - where 90.2% of agricultural land is also arable - shows a higher NPCi (median: 15.1; mean: 16.6 ± 9.2). While still low relative to more diverse landscapes, Emsland’s slightly elevated NPCi reflects a more mixed production system that includes significant dairy farming and livestock operations. These contribute to greater land-use variation and the retention of semi-natural elements within the agricultural matrix, partially mitigating the constraints of intensive arable cultivation.

**Fig. 12:**
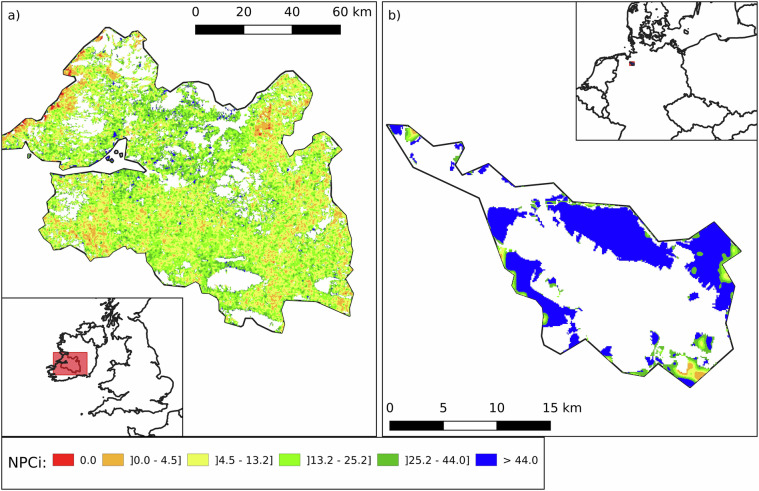
Spatial distribution of the Natural Pest Control index (NPCi) in two European regions: the Mid-West region of Ireland (NUTS3: IE051) on the left (panel a) and the Bremen - Kreisfreie Stadt region in northern Germany (NUTS3: DE501) on the right (panel b). The maps illustrate varying capacities to support natural pest control services, with colour gradients representing NPCi values. The Mid-West region of Ireland has 89.1% of its agricultural land dedicated to arable farming, of which only 0.2% is classified as extensively managed grassland (Herbaceous Areal category), and shows an NPCi with a median value of 14.3 and a mean of 16.0 ± 9.8. In contrast, the Bremen - Kreisfreie Stadt region has 91.1% of its agricultural land dedicated to grasslands and pastures, with 66.1% classified as extensively managed grassland (Herbaceous Areal category), and exhibits an NPCi with a median value of 69.4 and a mean of 63.0 ± 20.9. White areas indicate no data (non-agricultural land, outside the scope of the NPCi calculation).

Following arable land, pasture dominated areas exhibit the second lowest NPCi values across Europe. Ireland, which has the highest share of pasture among agriculturally significant countries, allocates 82.0% of its agricultural land to permanent grassland (NPCi median: 14.5; mean: 16.4 ± 10.6). Within Ireland, the Mid-West region (NUTS3: IE051; Fig. 12a) is even more pasture-intensive, with 89.1% of its agricultural land under permanent pasture. This region underpins Ireland’s strong livestock sector - including dairy, beef, and sheep farming - by providing high-quality forage. However, only 0.2% of its pasture area is classified as extensively managed grassland (HA), meaning that it offers limited SNH value for NE. This explains the region’s relatively low NPCi (median: 14.3; mean: 16.0 ± 9.8), despite its agronomic productivity.

In contrast, the Bremen - Kreisfreie Stadt region (NUTS3: DE501; Fig. 12b) in northern Germany, though largely urban, is surrounded by agricultural zones with extensive grasslands. These areas support dairy and beef farming, and importantly, 66.1% of Bremen’s agricultural land qualifies as extensively managed grassland (HA), the highest HA share among all analysed regions. This significant presence of herbaceous SNH contributes to a markedly higher NPCi (median: 69.4; mean: 63.0 ± 20.9), illustrating how pasture-based systems can enhance NPC potential when they incorporate low intensity management and habitat rich landscapes.

**Fig. 13:**
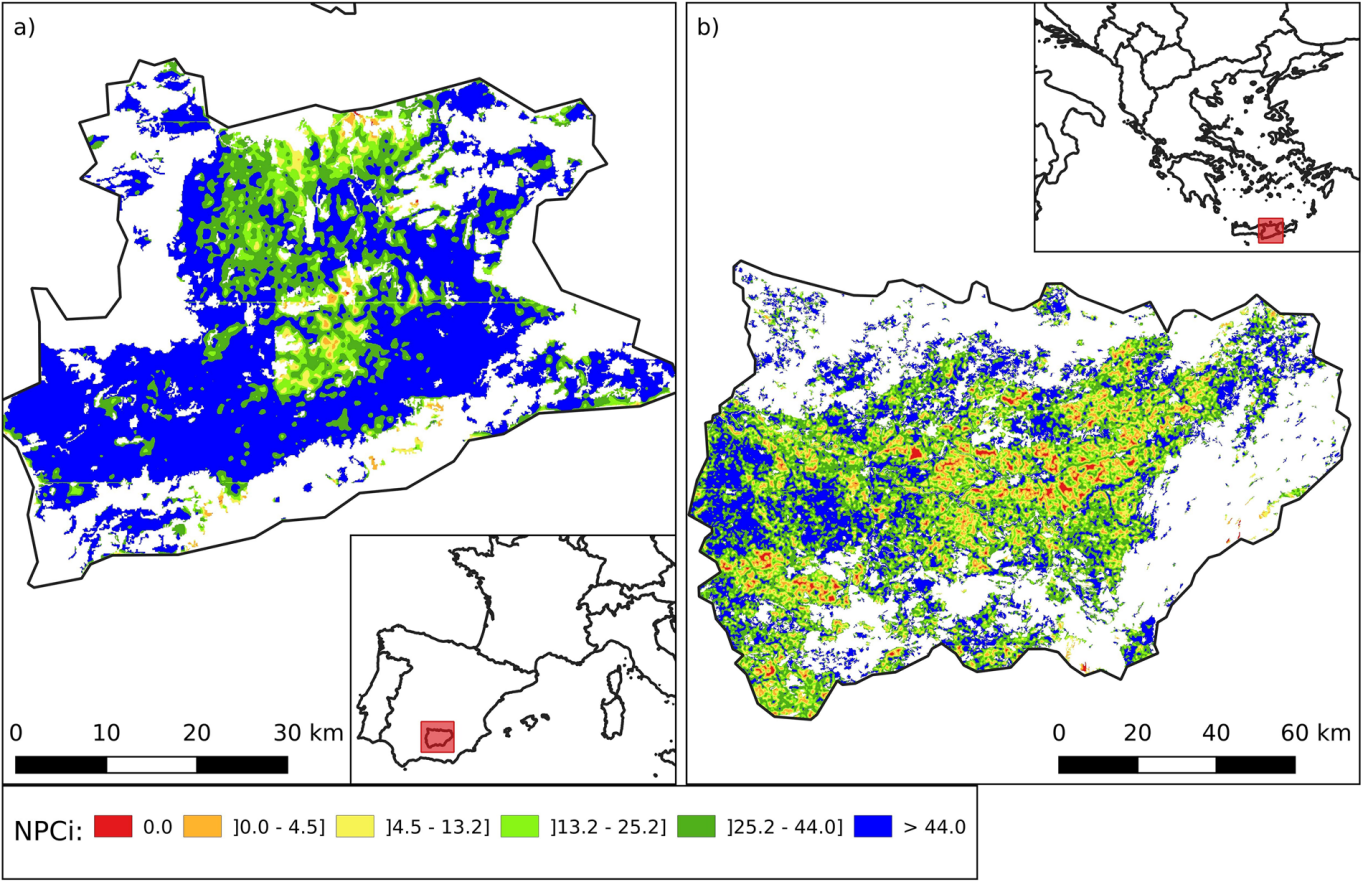
Spatial distribution of the Natural Pest Control index (NPCi) in two European regions: Heraklion in Greece (NUTS3: EL431) on the left (panel a) and Jaén in Spain (NUTS3: ES616) on the right (panel b). The maps illustrate varying capacities to support natural pest control services in regions with high proportions of permanent crops, with colour gradients representing NPCi values. Heraklion has 71.5% of its agricultural land dedicated to permanent crops, mainly olive groves, and exhibits a high NPCi with a median value of 52.4 and a mean of 48.3 ± 16.3. Jaén has 79.1% of its agricultural land cover with olive groves and shows a lower NPCi with a median value of 32.7and a mean of 31.7 ± 17.5. White areas indicate no data (non-agricultural land, outside the scope of the NPCi calculation).

Permanent crops exhibit the second highest NPCi values among agricultural land types. They are particularly prevalent in Greece, which leads Europe with 20.5% of its agricultural land under permanent crops, 16.1% of which is dedicated to olive groves (NPCi median: 43.4; mean: 39.9 ± 21.7). Within Greece, the region of Heraklion (NUTS3: EL431; Fig. 13a), located on the island of Crete, is a prime example. Olive groves cover 71.5% of Heraklion’s agricultural land, and the region benefits from favourable climatic and soil conditions. Traditional low-intensity farming practices, including manual olive harvesting and the retention of interstitial vegetation, contribute to a structurally diverse landscape that supports beneficial NE. These features are reflected in Heraklion’s relatively high NPCi (median: 52.4; mean: 48.3 ± 16.3).

In contrast, Jaén (NUTS3: ES616; Fig. 13b), located in Andalusia, Spain, also has a very high share of agricultural land under olives (79.1%) and is the largest olive producing province in the world, with more than 600,000 hectares of groves. Despite its scale, Jaén registers a substantially lower NPCi (median: 32.7; mean: 31.7 ± 17.5). This disparity likely reflects more intensive management, landscape homogenization, and reduced ground cover diversity within the groves, which can limit habitat availability for NE. These contrasting cases underscore that the NPCi of permanent crop systems depends not only on crop type, but also on how the land is managed and embedded within the broader landscape matrix. Disentangling these factors is critical for understanding and improving the ecological performance of perennial cropping systems.

**Fig. 14:**
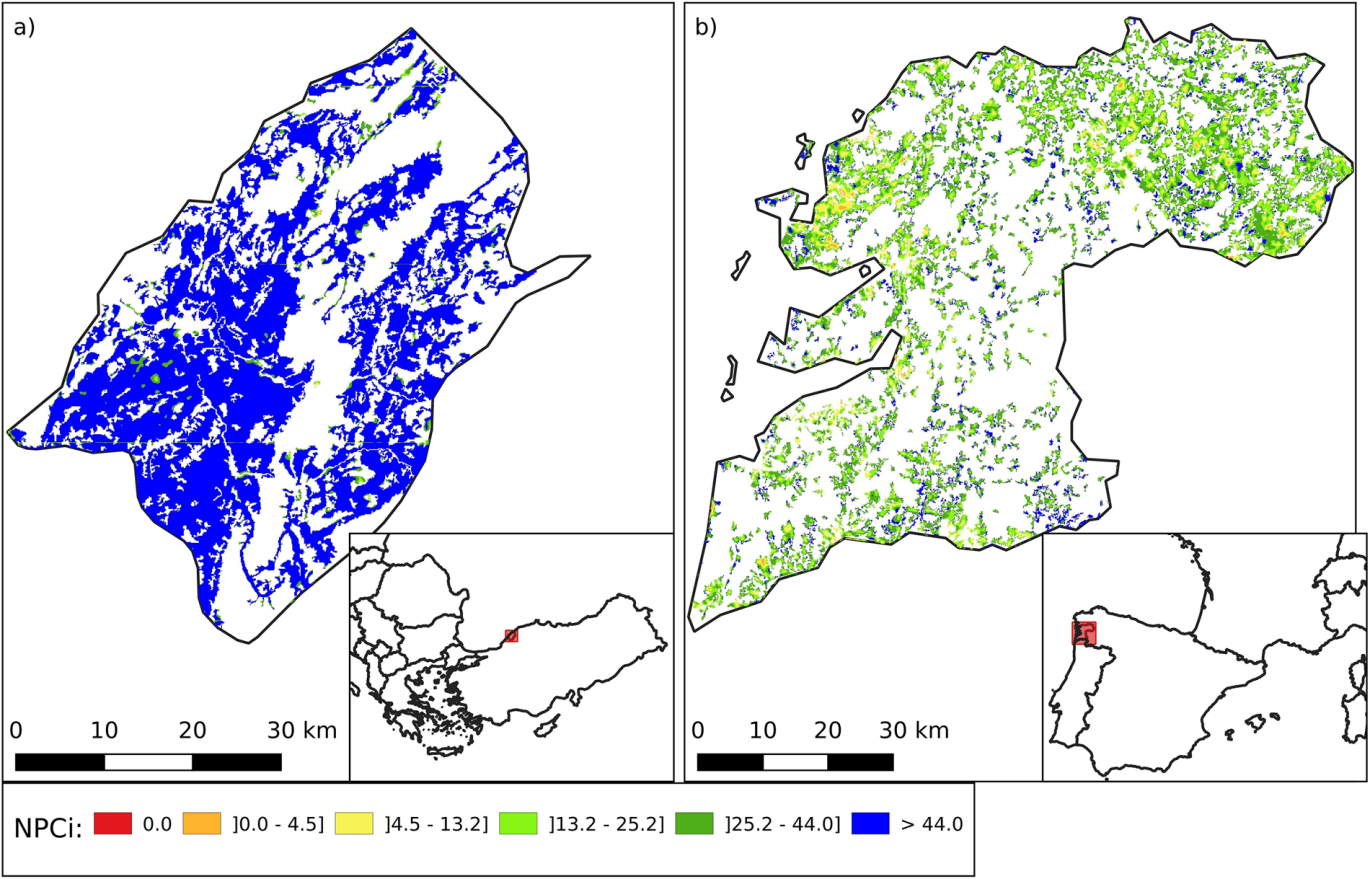
Spatial distribution of the Natural Pest Control index (NPCi) in two European regions: Ordu in Turkey (NUTS3: TR902) on the left and Aveyron in southern France (NUTS3: FRJ22) on the right. The maps illustrate varying capacities to support natural pest control services, with colour gradients representing NPCi values. Ordu (left) has an NPCi with a median value of 39.52 and a mean of 36.59 ± 17.96. Aveyron (right) has an NPCi with a median value of 22.46 and a mean of 25.44 ± 18.51. These maps provide a visual comparison of the potential for natural pest control in heterogeneous agricultural. White areas indicate no data (non-agricultural land, outside the scope of the NPCi calculation).

Regions with a high proportion of heterogeneous agricultural areas often exhibit elevated NPCi values, due to their structural complexity and lower management intensity. Bartın (NUTS3: TR813; Fig. 14a) in northern Turkey and Pontevedra (NUTS3: ES114; Fig. 14b) in northwestern Spain exemplify contrasting outcomes within this category. Heterogeneous land typically combines croplands with permanent crops, pastures, and interspersed semi-natural elements such as forest patches or shrublands. These mosaics support diverse habitat types and facilitate the movement and persistence of NE.

Bartın’s agricultural land is predominantly classified as heterogeneous (88.0%) and includes a well-integrated mix of arable fields, orchards, pastures, and surrounding forests. This rich landscape matrix contributes to Bartın achieving one of the highest NPCi scores in Europe (median: 65.8; mean: 64.6 ± 11.4). In contrast, Pontevedra, although classified as 97.7% heterogeneous agricultural land, shows a much lower NPCi (median: 29.5; mean: 29.7 ± 12.0). This discrepancy highlights that a high share of heterogeneous land alone does not guarantee high NPC potential. The ecological performance of such areas ultimately depends on the composition, spatial arrangement, and connectivity of their constituent elements. Where natural features are fragmented or poorly embedded in the landscape, the capacity to support NE may remain limited despite nominal land-use diversity.

## Usage Notes

Users can download and process the NPCi raster layers using standard GIS (ArcGIS, or open-source software tools such as QGIS, the raster and terra packages in R). At continental scale, the dataset requires approximately 1 GB of RAM, so users with limited memory are advised to clip the rasters to national or NUTS-3 boundaries to improve performance. Each pixel value ranges from 0 to 1 and represents the potential, not the realised rate, of NPC, as determined by the extent and configuration of surrounding SNH.

Because potential alone does not ensure field-level pest suppression, the map should be interpreted together with locally specific datasets such as crop type, pesticide application records, weather data, or field-based counts of pests and NE. Users should also be aware that SNH can provide resources for certain polyphagous herbivorous insects as well as NE, meaning that high values may not always translate into direct reductions in pest pressure. When applying the NPCi in planning or management contexts, it is therefore important to account for these trade-offs and to use complementary ecological information to support validation with ground observations.

It is also important to note that the underlying Copernicus High-Resolution Layers refer to the 2018 reference year. Any subsequent land-use changes - such as habitat restoration, degradation, or reclassification - are not captured and may lead to local over- or underestimation of SNH extent. For applications requiring greater temporal or thematic precision, users are encouraged to inspect the source imagery or substitute higher-resolution national land-cover datasets where available.

The NPCi specifically targets flying predator and parasitoid guilds and does not account for ground-dwelling NE, alternative prey dynamics, pesticide pressure, or climatic influences. Moreover, the coefficients used in the model were calibrated in temperate arable systems; applying the index to perennial crops or regions beyond Europe may require recalibration with local empirical data. However, the dataset’s fully scripted and modular workflow makes such adaptations straightforward. By substituting updated Copernicus HRLs or compatible land-cover data and adjusting the reference year in the configuration file, users can reapply the methodology in other spatial or temporal contexts.

Finally, the same SNH elements that support NPC also underpin other ES including pollination, soil conservation, water regulation, and carbon sequestration. The NPCi layer can therefore be integrated into broader ES assessments to support multifunctional landscape planning that benefits both agricultural productivity and environmental sustainability. When using or adapting the dataset, please cite the associated DOI and refer to the accompanying metadata, licence terms (CC-BY 4.0, compatible with the Copernicus EMS Open Licence), and detailed file descriptions Table 3.

**Table 3:** Comparative analysis of Natural Pest Control index (NPCi) values across pan-European countries.

Country	Median	Mean	SD	Min	Max	Percentile 25	Percentile 75
Albania	48.6	45.7	19.2	0	99.5	31.6	60.6
Austria	19	21	15.3	0	89.5	7.8	31.9
Belgium	13.3	16.1	12.4	0	84.5	6	23.9
Bulgaria	19	26.2	23.5	0	97.6	5.8	43.9
Croatia	39.9	38.9	23.2	0	96	18.6	58
Cyprus	30.5	30.6	20.3	0	84.5	12.8	46.7
Czech Republic	15.5	18.8	15.8	0	94.2	6	28
Denmark	11.1	13.8	10.6	0	93.1	5.8	19.3
Estonia	26.6	29.3	18.9	0	95	14.3	41.8
Finland	28.7	29.1	15.3	0	88	17.8	39.2
France	16.1	18.9	15	0	98.3	7	27.7
Germany	12.1	14.7	12.1	0	95.2	5	21.7
Greece	43.4	39.9	21.7	0	98.3	21.5	57.7
Hungary	15	21.3	20.3	0	92.8	4.5	33
Iceland	35.7	36.2	19	0	85	21.5	50.3
Ireland	14.5	16.4	10.6	0	91.8	8.6	22.4
Italy	31	32.2	21.6	0	99.4	12.4	49.9
Latvia	43.1	41.2	21.4	0	91	23.5	59.2
Liechtenstein	58	57.7	14.2	20.1	90.4	46.9	69.3
Lithuania	11.3	15.2	14.1	0	87.2	3.6	23.3
Luxembourg	14.3	16	11.1	0	65.2	6.7	23.5
Malta	10.2	13.6	11.1	0	69	5.6	18.5
Montenegro	68	64.4	17.6	0	99	56	77.1
Netherlands	8.9	11.7	10.6	0	79.2	3.2	17.5
North Macedonia	51.6	48.7	21.6	0	97	32.5	65.5
Norway	53.6	50.9	17.5	0	96.4	39.5	64.1
Poland	12.2	17.3	16.8	0	96.6	4.2	25.4
Portugal	31.6	32.4	19.4	0	96.8	16.7	46.8
Romania	15.2	26.9	28.5	0	98.4	0.5	51.7
Serbia	29	30.9	26.2	0	98.1	3.4	54.7
Slovakia	23.6	31.9	27.2	0	97.5	7.2	56.9
Slovenia	30.6	29.2	15.5	0	84.9	17.1	40.8
Spain	12.7	19.2	19.1	0	94.8	1.8	33.6
Sweden	25.1	25.9	16.8	0	91	11.6	37.7
Switzerland	59.3	55.2	19.4	0	96.3	41.8	71
Turkey	34.2	34.6	24.7	0	100	10.9	55.5
United Kingdom	12.8	15.8	13.2	0	99	6.3	21.8

The table presents the median, mean, standard deviation (sd), minimum (min), maximum (max), and the 25th and 75th percentiles of NPCi values for each country, highlighting the variability and potential for natural pest control services.

## Supplementary information

Supplementary Information

## Data Availability

The dataset supporting this study is openly available through the European Commission’s Joint Research Centre (JRC) Big Data Analytics Platform, and can be accessed at: https://jeodpp.jrc.ec.europa.eu/ftp/jrc-opendata/NPCI2018/.

## Code availability

The dataset supporting this study^27^ includes a detailed processing pipeline implemented using Python scripts and geospatial analysis tools. The pseudo-code outlining the processing steps is provided in the supplementary material under Supplementary Information 1–4 and is linked to the specific Python scripts. Each script is fully documented and includes an introductory section that provides an overview of the code’s workflow, detailing the sequence of operations and the purpose of each step. Inline comments are embedded throughout the code to enhance clarity, offering precise explanations for key operations. This comprehensive documentation ensures the scripts are user-friendly, facilitating reproducibility and adaptation. The processing pipeline utilizes the following components:

• WVM_TCD_script.py: combines and processes input raster layers for Woody Vegetation Mask (WVM) and Tree Cover Density (TCD) (Supplementary Method 1).

• MSPA Using GuidosToolbox^45^: conducts Morphological Spatial Pattern Analysis (MSPA) on the raster file WVM_TCD.tif to identify spatial patterns, such as core areas, edges, and corridors. This step involves input preparation, binary conversion, parameter selection, and output generation (Supplementary Method 2).

• SNH_script.py: generates the Semi-Natural Habitat (SNH) layer by combining MSPA results, extensive grasslands data, and CORINE land cover information (Supplementary Method 3).

• NPCi_script.py: computes the NPCi using spatial analysis of SNH layers, applying focal statistics and normalization techniques (Supplementary Method 4).
