# Supplementary material for: Mapping the potential of Natural Pest Control services in pan-European landscapes at 50 m resolution
Source: Sci Data. 2025 Nov 25;12:1865. doi: 10.1038/s41597-025-06138-7 (PMC12647132; doi:10.1038/s41597-025-06138-7)
Supplement: Supplementary file 1 — Supplementary Information [file 41597_2025_6138_MOESM1_ESM.docx]

Mapping the potential of Natural Pest Control services in pan-European landscapes at 50m resolution

Rui Catarino^1^, Ana Klinnert^2^, Ana Luisa Barbosa^2^, Raphael d’Andrimont^3^, Thomas Fellmann^2^, Renate Koeble^4^, Maria Luisa Paracchini^1^, Jean-Michel Terres^1^, Marijn van der Velde^1^, Peter Vogt^1^, Carlo Rega^1^

^1^European Commission, Joint Research Centre (JRC), Ispra, Italy

^2^European Commission, Joint Research Centre (JRC), Seville, Spain

^3^European Commission, Directorate-General Agriculture and Rural Development, Brussels, Belgium

^4^ARHS Developments S.A., Belvaux, Luxembourg

# Supplementary tables

Supplementary Table 1. CORINE Land Cover (CLC) nomenclature used in the NPC index (Source: <https://land.copernicus.eu/content/corine-land-cover-nomenclature-guidelines/html/>). This table outlines the CORINE Land Cover classification levels and definitions used in the Natural Pest Control (NPC) index. It details various types of agricultural areas, including arable land, permanent crops, pastures, and heterogeneous agricultural areas, providing specific descriptions for each land use category.

| Level 1 | Level 2 | Level 3 | Definition |
| --- | --- | --- | --- |
| 2. Agricultural areas | 2.1 Arable land | 2.1.1 Non-irrigated arable land | Cultivated land parcels under rainfed agricultural use for annually harvested non-permanent crops, normally under a crop rotation system, including fallow lands within such crop rotation. Fields with sporadic sprinkler-irrigation with non-permanent devices to support dominant rainfed cultivation are included. |
|  |  | 2.1.2 Permanently irrigated land | Cultivated land parcels under agricultural use for arable crops that are permanently or periodically irrigated, using a permanent infrastructure (irrigation channels, drainage network and additional irrigation facilities). Most of these crops cannot be cultivated without artificial water supply. Does not include sporadically irrigated land. |
|  |  | 2.1.3 Rice fields | Cultivated land parcels prepared for rice production, consisting of periodically flooded flat surfaces with irrigation channels. |
|  | 2.2 Permanent Crops | Vineyards | Areas planted with vines, vineyard parcels covering >50% and determining the land use of the area. |
|  |  | Fruit trees and berry plantations | Cultivated parcels planted with fruit trees and shrubs, intended for fruit production, including nuts. The planting pattern can be by single or mixed fruit species, both in association with permanently grassy surfaces. |
|  |  | Olive groves | Cultivated areas planted with olive trees. |
|  | 2.3 Pastures | 2.3.1 Pastures | Lands that are permanently used (at least 5 years) for fodder production. Includes natural or sown herbaceous species, unimproved or lightly improved meadows and grazed or mechanically harvested meadows. Regular agriculture impact influences the natural development of natural herbaceous species composition. |
|  | 2.4 Heterogeneous agricultural areas | 2.4.1 Annual crops associated with permanent crops | Cultivated land parcels with non-permanent crops (mostly arable land) associated with permanent crops (fruit trees or olive trees or vines) on the same parcel. |
|  |  | 2.4.2 Complex cultivation patterns | Mosaic of small cultivated land parcels with different cultivation types -annual crops, pasture and/or permanent crops-, eventually with scattered houses or gardens. |
|  |  | 2.4.3 Land principally occupied by agriculture, with significant areas of natural vegetation | Areas principally occupied by agriculture, interspersed with significant natural or semi-natural areas (including forests, shrubs, wetlands, water bodies, mineral outcrops) in a mosaic pattern. |
|  |  | 2.4.4 Agro-forestry areas | Annual crops or grazing land under the wooded cover of forestry species. |

Supplementary Table 2. Linear Model Analysis of Natural Pest Control Index (NPCi) and Semi-Natural Habitat (SNH) Share for Various Countries. This table presents the results of a linear model analysis, with the intercept forced to pass through the origin (NPCi ~ SNH). The Natural Pest Control index (NPCi) ranges from 0-100, while the Semi-Natural Habitat share (SNH) is expressed as a percentage from 0-100%. For each country, the table lists the estimated coefficient, standard error, test statistic, degrees of freedom (df), p-value, significance level, and R-squared value. Significance codes: *** p < 0.001, ** p < 0.01, * p < 0.05, ns: not significant. The linear model was forced through the origin because it is logical to assume that, according to the available data, no semi-natural habitat should correspond to no natural pest control, reflecting the theoretical expectation that natural habitats are necessary for pest control. This approach aligns the model more closely with biological realities and simplifies the interpretation of the results.

| Country | estimate | std.error | statistic | df | p.value | significance | rsquared |
| --- | --- | --- | --- | --- | --- | --- | --- |
| Albania | 4.735 | 0.778 | 6.086 | 11 | 0 | *** | 0.771 |
| Austria | 6.575 | 0.833 | 7.895 | 34 | 0 | *** | 0.647 |
| Belgium | 3.123 | 0.222 | 14.056 | 43 | 0 | *** | 0.821 |
| Bulgaria | 3.042 | 0.241 | 12.628 | 27 | 0 | *** | 0.855 |
| Croatia | 3.326 | 0.39 | 8.52 | 20 | 0 | *** | 0.784 |
| Cyprus | 5.753 |  |  | 0 |  | ns | 1 |
| Czech Republic | 3.602 | 0.154 | 23.466 | 13 | 0 | *** | 0.977 |
| Denmark | 3.191 | 0.538 | 5.929 | 10 | 0 | *** | 0.779 |
| Estonia | 6.459 | 1.326 | 4.871 | 4 | 0.008 | ** | 0.856 |
| Finland | 14.573 | 2.684 | 5.429 | 18 | 0 | *** | 0.621 |
| France | 3.261 | 0.21 | 15.493 | 92 | 0 | *** | 0.723 |
| Germany | 4.107 | 0.098 | 42.03 | 400 | 0 | *** | 0.815 |
| Greece | 3.79 | 0.334 | 11.339 | 49 | 0 | *** | 0.724 |
| Hungary | 2.77 | 0.197 | 14.063 | 19 | 0 | *** | 0.912 |
| Iceland | 43.279 | 6.132 | 7.058 | 1 | 0.09 | ns | 0.98 |
| Ireland | 3.158 | 0.293 | 10.787 | 7 | 0 | *** | 0.943 |
| Italy | 2.265 | 0.143 | 15.838 | 106 | 0 | *** | 0.703 |
| Latvia | 3.952 | 0.798 | 4.955 | 5 | 0.004 | ** | 0.831 |
| Liechtenstein | 6.832 |  |  | 0 |  | ns | 1 |
| Lithuania | 3.479 | 0.261 | 13.33 | 9 | 0 | *** | 0.952 |
| Luxembourg | 4.208 |  |  | 0 |  | ns | 1 |
| Malta | 3.445 | 0.287 | 12 | 1 | 0.053 | ns | 0.993 |
| Montenegro | 9.728 |  |  | 0 |  | ns | 1 |
| Netherlands | 2.688 | 0.156 | 17.27 | 39 | 0 | *** | 0.884 |
| North Macedonia | 3.471 | 0.496 | 7.004 | 7 | 0 | *** | 0.875 |
| Norway | 20.779 | 4.47 | 4.649 | 10 | 0.001 | ** | 0.684 |
| Poland | 3.124 | 0.158 | 19.794 | 72 | 0 | *** | 0.845 |
| Portugal | 2.966 | 0.478 | 6.209 | 24 | 0 | *** | 0.616 |
| Romania | 2.614 | 0.124 | 21.107 | 41 | 0 | *** | 0.916 |
| Serbia | 2.691 | 0.209 | 12.872 | 24 | 0 | *** | 0.873 |
| Slovakia | 3.277 | 0.275 | 11.92 | 7 | 0 | *** | 0.953 |
| Slovenia | 4.895 | 0.49 | 9.998 | 11 | 0 | *** | 0.901 |
| Spain | 1.666 | 0.241 | 6.9 | 58 | 0 | *** | 0.451 |
| Sweden | 9.335 | 1.914 | 4.877 | 20 | 0 | *** | 0.543 |
| Switzerland | 3.522 | 0.41 | 8.591 | 25 | 0 | *** | 0.747 |
| Turkey | 3.208 | 0.163 | 19.635 | 80 | 0 | *** | 0.828 |
| United Kingdom | 3.62 | 0.217 | 16.654 | 168 | 0 | *** | 0.623 |

Supplementary Table 3. Distribution of Semi-Natural Habitats (SNH) and Agricultural Land in Various European Countries. This table shows the area (km²), national share (%), and agricultural share (%) of different semi-natural habitats (SNH) types in various European countries. The SNH types are Herbaceous Areal (HA), Woody Areal – edge (WAe), Woody Areal – interior (WAi), and Woody Linear (WL). AgLand represents the main agricultural land classes.

|  | Area km2 | | | | | National share (%) | | | | | Agricultural share (%) | | | |
| --- | --- | --- | --- | --- | --- | --- | --- | --- | --- | --- | --- | --- | --- | --- |
| Country | AgLand | HA | WAe | WAi | WL | AgLand | HA | WAe | WAi | WL | HA | WAe | WAi | WL |
| Albania | 8053.6 | 2295.3 | 699.5 | 978.2 | 233.6 | 28 | 8 | 2.4 | 3.4 | 0.8 | 28.5 | 8.7 | 12.1 | 2.9 |
| Austria | 26782 | 456.9 | 1695.3 | 2045.8 | 513.6 | 31.9 | 0.5 | 2 | 2.4 | 0.6 | 1.7 | 6.3 | 7.6 | 1.9 |
| Belgium | 17490.1 | 47.7 | 995.9 | 1313.4 | 305.8 | 57 | 0.2 | 3.2 | 4.3 | 1 | 0.3 | 5.7 | 7.5 | 1.7 |
| Bulgaria | 57349.2 | 7875.2 | 3053.3 | 5108.8 | 947.4 | 51.7 | 7.1 | 2.8 | 4.6 | 0.9 | 13.7 | 5.3 | 8.9 | 1.7 |
| Croatia | 22364.4 | 5078.1 | 1724.5 | 2438.4 | 608.5 | 39.6 | 9 | 3.1 | 4.3 | 1.1 | 22.7 | 7.7 | 10.9 | 2.7 |
| Cyprus | 4395.4 | 1048.5 | 196.1 | 200.3 | 63.5 | 47.4 | 11.3 | 2.1 | 2.2 | 0.7 | 23.9 | 4.5 | 4.6 | 1.4 |
| Czech Republic | 44821.9 | 1314.8 | 2494 | 2947.3 | 810.3 | 56.8 | 1.7 | 3.2 | 3.7 | 1 | 2.9 | 5.6 | 6.6 | 1.8 |
| Denmark | 31702.5 | 67.8 | 1631.3 | 1594.5 | 614.7 | 72.6 | 0.2 | 3.7 | 3.7 | 1.4 | 0.2 | 5.1 | 5 | 1.9 |
| Estonia | 14305.5 | 1450.2 | 955.9 | 1407.7 | 297.1 | 31.5 | 3.2 | 2.1 | 3.1 | 0.7 | 10.1 | 6.7 | 9.8 | 2.1 |
| Finland | 27938.3 | 1822.5 | 2077.9 | 3057.6 | 514.6 | 8.3 | 0.5 | 0.6 | 0.9 | 0.2 | 6.5 | 7.4 | 10.9 | 1.8 |
| France | 323725.2 | 7387.1 | 19650.5 | 20564.7 | 8142.2 | 50.7 | 1.2 | 3.1 | 3.2 | 1.3 | 2.3 | 6.1 | 6.4 | 2.5 |
| Germany | 204760.9 | 1716.1 | 9927.1 | 10118.9 | 3520.4 | 57.2 | 0.5 | 2.8 | 2.8 | 1 | 0.8 | 4.8 | 4.9 | 1.7 |
| Greece | 50969.2 | 8248.4 | 4740.4 | 9706.2 | 1526.9 | 38.7 | 6.3 | 3.6 | 7.4 | 1.2 | 16.2 | 9.3 | 19 | 3 |
| Hungary | 60382.7 | 7976.3 | 2206.1 | 2609.2 | 707.2 | 64.9 | 8.6 | 2.4 | 2.8 | 0.8 | 13.2 | 3.7 | 4.3 | 1.2 |
| Iceland | 2573.2 | 1312.2 | 14.4 | 6.3 | 4.8 | 2.5 | 1.3 | 0 | 0 | 0 | 51 | 0.6 | 0.2 | 0.2 |
| Ireland | 47096.2 | 140.2 | 3070.1 | 1873 | 1664 | 67.4 | 0.2 | 4.4 | 2.7 | 2.4 | 0.3 | 6.5 | 4 | 3.5 |
| Italy | 156351.2 | 18164.3 | 12252.3 | 24628.8 | 4152.9 | 52 | 6 | 4.1 | 8.2 | 1.4 | 11.6 | 7.8 | 15.8 | 2.7 |
| Latvia | 25553.3 | 7435.4 | 1663.8 | 2060.9 | 487.5 | 39.6 | 11.5 | 2.6 | 3.2 | 0.8 | 29.1 | 6.5 | 8.1 | 1.9 |
| Liechtenstein | 31.8 | 17.7 | 2.4 | 1.6 | 1.1 | 20 | 11.1 | 1.5 | 1 | 0.7 | 55.7 | 7.5 | 5 | 3.5 |
| Lithuania | 38184.4 | 853 | 1688.7 | 2006.2 | 484.7 | 58.8 | 1.3 | 2.6 | 3.1 | 0.7 | 2.2 | 4.4 | 5.3 | 1.3 |
| Luxembourg | 1363.3 | 3 | 65.5 | 83.4 | 15.4 | 52.5 | 0.1 | 2.5 | 3.2 | 0.6 | 0.2 | 4.8 | 6.1 | 1.1 |
| Malta | 162.1 | 4.6 | 9.6 | 4 | 3.3 | 51.7 | 1.5 | 3.1 | 1.3 | 1.1 | 2.8 | 5.9 | 2.5 | 2 |
| Montenegro | 2226.4 | 907.2 | 266.3 | 502.6 | 88.9 | 16.1 | 6.5 | 1.9 | 3.6 | 0.6 | 40.7 | 12 | 22.6 | 4 |
| Netherlands | 23840.1 | 34.8 | 1074.5 | 814.3 | 516.1 | 63.8 | 0.1 | 2.9 | 2.2 | 1.4 | 0.1 | 4.5 | 3.4 | 2.2 |
| North Macedonia | 9135.4 | 3299.1 | 659.9 | 913.6 | 246.4 | 35.9 | 13 | 2.6 | 3.6 | 1 | 36.1 | 7.2 | 10 | 2.7 |
| Norway | 16401.7 | 4601.3 | 1809 | 3291.8 | 559.1 | 4.2 | 1.2 | 0.5 | 0.9 | 0.1 | 28.1 | 11 | 20.1 | 3.4 |
| Poland | 183522.6 | 7234.4 | 9186.3 | 10472.2 | 2868.7 | 58.8 | 2.3 | 2.9 | 3.4 | 0.9 | 3.9 | 5 | 5.7 | 1.6 |
| Portugal | 44027.2 | 2365.9 | 4291 | 7708.5 | 1722.6 | 47.9 | 2.6 | 4.7 | 8.4 | 1.9 | 5.4 | 9.7 | 17.5 | 3.9 |
| Romania | 135415.5 | 28056.7 | 5172.5 | 7941.4 | 1516.7 | 56.8 | 11.8 | 2.2 | 3.3 | 0.6 | 20.7 | 3.8 | 5.9 | 1.1 |
| Serbia | 42732.7 | 7553.9 | 2629.3 | 4286.6 | 737.3 | 55.1 | 9.7 | 3.4 | 5.5 | 1 | 17.7 | 6.2 | 10 | 1.7 |
| Slovakia | 23122.9 | 4925.2 | 1300.7 | 1856.5 | 405.2 | 47.2 | 10 | 2.7 | 3.8 | 0.8 | 21.3 | 5.6 | 8 | 1.8 |
| Slovenia | 6933.9 | 112.1 | 658.1 | 1031.2 | 187.9 | 34.2 | 0.6 | 3.2 | 5.1 | 0.9 | 1.6 | 9.5 | 14.9 | 2.7 |
| Spain | 242660.8 | 1072.5 | 13806.9 | 41911.2 | 4629.4 | 47.9 | 0.2 | 2.7 | 8.3 | 0.9 | 0.4 | 5.7 | 17.3 | 1.9 |
| Sweden | 39668.4 | 1480.7 | 3032.8 | 4146.7 | 927.8 | 8.8 | 0.3 | 0.7 | 0.9 | 0.2 | 3.7 | 7.6 | 10.5 | 2.3 |
| Switzerland | 11314.2 | 5595.2 | 737.2 | 868.3 | 197.4 | 27.4 | 13.6 | 1.8 | 2.1 | 0.5 | 49.5 | 6.5 | 7.7 | 1.7 |
| Turkey | 340464.9 | 83426.4 | 16536.8 | 28970.1 | 6362.6 | 43.6 | 10.7 | 2.1 | 3.7 | 0.8 | 24.5 | 4.9 | 8.5 | 1.9 |
| United Kingdom | 136123.8 | 3212.2 | 7515.7 | 5914 | 3200.6 | 55.7 | 1.3 | 3.1 | 2.4 | 1.3 | 2.4 | 5.5 | 4.3 | 2.4 |

Supplementary Table 4. Comparative analysis of the Natural Pest Control Index (NPCi) values across pan-European NUTS1 regions. The table presents the median, mean, standard deviation (SD), minimum (min), maximum (max), and the 25^th^ and 75^th^ percentiles of NPCi values for each NUTS 1 region.

| **Region** | **Name** | **Median** | **Mean** | **SD** | **Min** | **Max** | **p25** | **p75** |
| --- | --- | --- | --- | --- | --- | --- | --- | --- |
| AL0 | Shqipëria | 48.61 | 45.67 | 19.18 | 0 | 99.5 | 31.6 | 60.57 |
| AT1 | Ostösterreich | 11.56 | 15.49 | 13.88 | 0 | 86.76 | 3.76 | 24.6 |
| AT2 | Südösterreich | 31.37 | 30.34 | 13.41 | 0 | 86.95 | 20.74 | 40.09 |
| AT3 | Westösterreich | 19.74 | 22.24 | 15.14 | 0 | 89.49 | 10.64 | 30.84 |
| BE1 | Région de Bruxelles-Capitale/Brussels Hoofdstedelijk Gewest | 41.66 | 41.77 | 12.94 | 8.29 | 67.2 | 31.36 | 52.8 |
| BE2 | Vlaams Gewest | 13.52 | 16.71 | 12.74 | 0 | 75.15 | 6.48 | 24.43 |
| BE3 | Région wallonne | 13.19 | 15.6 | 12.11 | 0 | 84.47 | 5.52 | 23.45 |
| BG3 | Severna i Yugoiztochna Bulgaria | 13.09 | 20.39 | 20.5 | 0 | 97.64 | 3.87 | 31.76 |
| BG4 | Yugozapadna i Yuzhna tsentralna Bulgaria | 44.79 | 42.11 | 23.66 | 0 | 96.9 | 21.4 | 61.8 |
| CH0 | Schweiz/Suisse/Svizzera | 59.28 | 55.24 | 19.44 | 0 | 96.32 | 41.77 | 71 |
| CY0 | Kýpros | 30.53 | 30.63 | 20.31 | 0 | 84.53 | 12.83 | 46.72 |
| CZ0 | Česko | 15.53 | 18.85 | 15.85 | 0 | 94.16 | 5.99 | 27.99 |
| DE1 | Baden-Württemberg | 13.94 | 16.18 | 12.43 | 0 | 86.24 | 5.78 | 24.21 |
| DE2 | Bayern | 12.84 | 14.98 | 11.43 | 0 | 90.43 | 5.43 | 22.54 |
| DE3 | Berlin | 45.54 | 45.12 | 19.45 | 1.27 | 85.43 | 30.65 | 60.8 |
| DE4 | Brandenburg | 9.77 | 12.9 | 11.78 | 0 | 90.44 | 3.85 | 18.98 |
| DE5 | Bremen | 69 | 62.76 | 20.65 | 0 | 91.29 | 50.81 | 79.97 |
| DE6 | Hamburg | 54.14 | 52.54 | 19.01 | 2.91 | 89.1 | 39.23 | 67.21 |
| DE7 | Hessen | 15.27 | 17.47 | 12.98 | 0 | 90.98 | 6.92 | 25.57 |
| DE9 | Niedersachsen | 12.88 | 15.02 | 11.72 | 0 | 95.16 | 6 | 21.63 |
| DEA | Nordrhein-Westfalen | 14.76 | 17.01 | 12.54 | 0 | 90.37 | 7.38 | 24.32 |
| DEB | Rheinland-Pfalz | 15.37 | 17.27 | 12.91 | 0 | 86.55 | 6.39 | 25.98 |
| DEC | Saarland | 22.54 | 23.13 | 12.58 | 0 | 84.79 | 13.79 | 31.25 |
| DED | Sachsen | 11.26 | 13.87 | 11.65 | 0 | 88.41 | 4.31 | 20.86 |
| DEE | Sachsen-Anhalt | 6.44 | 10.14 | 11.06 | 0 | 89.26 | 1.88 | 15.11 |
| DEF | Schleswig-Holstein | 10.55 | 12.66 | 9.81 | 0 | 84.34 | 4.99 | 18.15 |
| DEG | Thüringen | 10.29 | 13.76 | 12.69 | 0 | 94.82 | 3.5 | 20.95 |
| DK0 | Danmark | 11.08 | 13.76 | 10.56 | 0 | 93.14 | 5.75 | 19.29 |
| EE0 | Eesti | 26.61 | 29.34 | 18.95 | 0 | 95.02 | 14.27 | 41.78 |
| EL3 | Attiki | 25.65 | 27.32 | 18.95 | 0 | 80.48 | 10.63 | 41.89 |
| EL4 | Nisia Aigaiou, Kriti | 49.59 | 46.23 | 17.98 | 0 | 95.4 | 34.97 | 59.51 |
| EL5 | Voreia Elláda | 34.15 | 34.34 | 21.48 | 0 | 97.18 | 14.98 | 52.33 |
| EL6 | Kentriki Elláda | 48.99 | 43.7 | 21.52 | 0 | 98.31 | 27.63 | 60.48 |
| ES1 | Noroeste | 31.03 | 30.83 | 13.74 | 0 | 89.68 | 20.75 | 40.81 |
| ES2 | Noreste | 4.73 | 11.84 | 15.25 | 0 | 88.36 | 0.24 | 18.63 |
| ES3 | Comunidad de Madrid | 10.99 | 16.91 | 17.23 | 0 | 90.29 | 2.76 | 26.34 |
| ES4 | Centro (ES) | 6.46 | 14.6 | 17.56 | 0 | 93.15 | 0.78 | 23.9 |
| ES5 | Este | 30.11 | 30.3 | 19.61 | 0 | 89.16 | 12.57 | 47 |
| ES6 | Sur | 24.23 | 25.79 | 19.86 | 0 | 90.09 | 6.65 | 42.19 |
| ES7 | Canarias | 12.51 | 22.5 | 24.12 | 0 | 94.8 | 2.29 | 37.72 |
| FI1 | Manner-Suomi | 28.63 | 29.01 | 15.23 | 0 | 88.02 | 17.73 | 39.07 |
| FI2 | Åland | 52.07 | 48.8 | 18.29 | 0 | 86.58 | 36.73 | 62.98 |
| FR1 | Ile-de-France | 6.38 | 10.52 | 11.51 | 0 | 78.77 | 1.24 | 16.57 |
| FRB | Centre — Val de Loire | 11.55 | 15.51 | 14.92 | 0 | 93.53 | 3.23 | 23.63 |
| FRC | Bourgogne-Franche-Comté | 15.15 | 17.55 | 13.44 | 0 | 94.05 | 7.06 | 25.25 |
| FRD | Normandie | 15.01 | 16.63 | 11.97 | 0 | 88.23 | 6.84 | 24.4 |
| FRE | Hauts-de-France | 6.89 | 10.37 | 10.75 | 0 | 85.2 | 1.58 | 16.12 |
| FRF | Grand Est | 7.41 | 10.87 | 11 | 0 | 98.27 | 1.84 | 17.02 |
| FRG | Pays de la Loire | 14.99 | 17.82 | 13.06 | 0 | 91.34 | 8.23 | 24.37 |
| FRH | Bretagne | 18.57 | 20.33 | 11.84 | 0 | 86.03 | 11.12 | 27.77 |
| FRI | Nouvelle-Aquitaine | 20.24 | 21.56 | 13.99 | 0 | 93.61 | 10.43 | 30.9 |
| FRJ | Occitanie | 22.74 | 25.53 | 16.23 | 0 | 94.59 | 12.91 | 35.32 |
| FRK | Auvergne-Rhône-Alpes | 21.68 | 23.67 | 14.95 | 0 | 91.53 | 11.97 | 33.19 |
| FRL | Provence-Alpes-Côte d’Azur | 41.75 | 40.98 | 18.3 | 0 | 96.13 | 27.53 | 54.24 |
| FRM | Corse | 52.39 | 50.63 | 16.36 | 0 | 95.19 | 40.21 | 62.64 |
| HR0 | Hrvatska | 39.87 | 38.87 | 23.22 | 0 | 95.96 | 18.59 | 57.97 |
| HU1 | Közép-Magyarország | 19.95 | 24.38 | 20.53 | 0 | 92.8 | 6.46 | 38.32 |
| HU2 | Dunántúl | 16.47 | 22.11 | 19.66 | 0 | 92.49 | 6.03 | 33.47 |
| HU3 | Alföld és Észak | 13.48 | 20.42 | 20.55 | 0 | 92.47 | 3.43 | 32.03 |
| IE0 | Ireland | 14.51 | 16.44 | 10.59 | 0 | 91.85 | 8.56 | 22.36 |
| IS0 | Ísland | 35.74 | 36.17 | 18.97 | 0 | 85.01 | 21.53 | 50.34 |
| ITC | Nord-Ovest | 19.74 | 25.92 | 20.26 | 0 | 90.5 | 8.59 | 42.28 |
| ITF | Sud | 36.78 | 35.58 | 21.3 | 0 | 99.41 | 17.2 | 52.45 |
| ITG | Isole | 35.97 | 35.88 | 20.94 | 0 | 95.39 | 18.2 | 52.8 |
| ITH | Nord-Est | 12.97 | 19.33 | 17.89 | 0 | 90.63 | 4.62 | 31.47 |
| ITI | Centro (IT) | 42.45 | 41.27 | 20.11 | 0 | 95.1 | 24.8 | 57.67 |
| LI0 | Liechtenstein | 57.96 | 57.73 | 14.18 | 20.07 | 90.37 | 46.94 | 69.27 |
| LT0 | Lietuva | 11.3 | 15.23 | 14.1 | 0 | 87.2 | 3.58 | 23.34 |
| LU0 | Luxembourg | 14.28 | 15.97 | 11.15 | 0 | 65.25 | 6.69 | 23.5 |
| LV0 | Latvija | 43.08 | 41.23 | 21.38 | 0 | 90.98 | 23.54 | 59.25 |
| ME0 | Crna Gora | 68.01 | 64.37 | 17.57 | 0 | 99 | 55.98 | 77.1 |
| MK0 | Severna Makedonija | 51.63 | 48.68 | 21.57 | 0 | 97.04 | 32.51 | 65.47 |
| MT0 | Malta | 10.24 | 13.61 | 11.12 | 0 | 69.01 | 5.6 | 18.47 |
| NL1 | Noord-Nederland | 6.73 | 9.96 | 9.88 | 0 | 77.51 | 2.11 | 15.12 |
| NL2 | Oost-Nederland | 11.57 | 13.76 | 10.79 | 0 | 77 | 5.18 | 19.96 |
| NL3 | West-Nederland | 5.18 | 8.22 | 9.15 | 0 | 75.95 | 1.77 | 11.35 |
| NL4 | Zuid-Nederland | 13.5 | 15.63 | 11.19 | 0 | 79.19 | 6.86 | 22.32 |
| NO0 | Norge | 53.65 | 50.92 | 17.47 | 0 | 96.45 | 39.52 | 64.12 |
| PL2 | Makroregion południowy | 20.31 | 22.44 | 16.37 | 0 | 93.13 | 9.02 | 32.83 |
| PL4 | Makroregion północno-zachodni | 9.83 | 15.08 | 15.95 | 0 | 91.94 | 3.06 | 22.01 |
| PL5 | Makroregion południowo-zachodni | 10 | 14.84 | 15.42 | 0 | 92.69 | 2.88 | 22.15 |
| PL6 | Makroregion północny | 10.56 | 14.09 | 12.66 | 0 | 90.48 | 4.03 | 21.13 |
| PL7 | Makroregion centralny | 11.66 | 16.02 | 14.78 | 0 | 88.92 | 3.92 | 24.58 |
| PL8 | Makroregion wschodni | 13.86 | 21.05 | 20.9 | 0 | 95.66 | 4.75 | 30.49 |
| PL9 | Makroregion województwo mazowieckie | 14.83 | 19.22 | 16.74 | 0 | 96.6 | 5.64 | 28.7 |
| PT1 | Continente | 31.13 | 31.73 | 18.81 | 0 | 95.96 | 16.41 | 46.02 |
| PT2 | Região Autónoma dos Açores | 57.9 | 53.16 | 25.63 | 0 | 96.76 | 31.56 | 76.31 |
| PT3 | Região Autónoma da Madeira | 63.07 | 59.83 | 15.43 | 0 | 86.52 | 53.53 | 70.55 |
| RO1 | Macroregiunea Unu | 57.89 | 51.52 | 26.21 | 0 | 98.01 | 29.74 | 74.29 |
| RO2 | Macroregiunea Doi | 6.49 | 19.56 | 24.54 | 0 | 98.42 | 0 | 35.06 |
| RO3 | Macroregiunea Trei | 0.53 | 8.61 | 15.94 | 0 | 92.51 | 0 | 8.87 |
| RO4 | Macroregiunea Patru | 15.36 | 25.38 | 26.24 | 0 | 97.49 | 1.07 | 47.15 |
| RS1 | Serbia - sever | 2.79 | 10.37 | 16.02 | 0 | 88.62 | 0.22 | 12.81 |
| RS2 | Serbia - jug | 51.17 | 48.27 | 19.75 | 0 | 98.06 | 34.66 | 63.21 |
| SE1 | Östra Sverige | 25.6 | 26.3 | 16.33 | 0 | 90.96 | 13.21 | 37.3 |
| SE2 | Södra Sverige | 20.74 | 22.44 | 16.03 | 0 | 89.25 | 8.07 | 34.63 |
| SE3 | Norra Sverige | 33.71 | 34.88 | 16.46 | 0 | 88.45 | 22.9 | 45.65 |
| SI0 | Slovenija | 30.6 | 29.18 | 15.46 | 0 | 84.91 | 17.1 | 40.83 |
| SK0 | Slovensko | 23.57 | 31.85 | 27.16 | 0 | 97.47 | 7.16 | 56.87 |
| TR1 | İstanbul | 35.94 | 35.99 | 21.24 | 0 | 90.74 | 17.38 | 53.63 |
| TR2 | Batı Marmara | 29.43 | 32.85 | 24.68 | 0 | 100 | 9.58 | 54.94 |
| TR3 | Ege | 36.91 | 35.56 | 22.66 | 0 | 94.89 | 14.57 | 54.76 |
| TR4 | Doğu Marmara | 40.19 | 37.72 | 22.96 | 0 | 96.37 | 17.63 | 56.35 |
| TR5 | Batı Anadolu | 19.1 | 27.04 | 25.28 | 0 | 100 | 4.39 | 46.08 |
| TR6 | Akdeniz | 36.1 | 34.68 | 22.68 | 0 | 96.86 | 13.32 | 53.46 |
| TR7 | Orta Anadolu | 22.89 | 28.13 | 23.61 | 0 | 96.61 | 6.76 | 46.03 |
| TR8 | Batı Karadeniz | 46.22 | 42.63 | 22.78 | 0 | 98.96 | 23.8 | 60.93 |
| TR9 | Doğu Karadeniz | 51.02 | 51 | 15.3 | 0 | 96.91 | 40.9 | 60.57 |
| TRA | Kuzeydoğu Anadolu | 51.44 | 48.15 | 23.84 | 0 | 96.56 | 29.03 | 68.53 |
| TRB | Ortadoğu Anadolu | 51.25 | 47.77 | 22.29 | 0 | 96.18 | 31.54 | 65.65 |
| TRC | Güneydoğu Anadolu | 14.55 | 22.89 | 23.23 | 0 | 96.41 | 1.81 | 40.62 |
| UKC | North East (England) | 10.42 | 12.61 | 9.44 | 0 | 99.04 | 5.3 | 17.95 |
| UKD | North West (England) | 12.44 | 14.2 | 9.63 | 0 | 78.23 | 6.71 | 19.92 |
| UKE | Yorkshire and the Humber | 8.44 | 10.82 | 9.16 | 0 | 84.24 | 3.7 | 15.66 |
| UKF | East Midlands (England) | 8.17 | 10.27 | 8.5 | 0 | 80.3 | 3.76 | 14.65 |
| UKG | West Midlands (England) | 12.02 | 13.76 | 9.09 | 0 | 74.33 | 6.79 | 19.02 |
| UKH | East of England | 9.64 | 11.78 | 9.39 | 0 | 84.29 | 4.54 | 16.88 |
| UKI | London | 51.35 | 49.58 | 20.14 | 0 | 89.4 | 34.86 | 66.22 |
| UKJ | South East (England) | 14.93 | 16.65 | 11.39 | 0 | 87.85 | 7.42 | 24.27 |
| UKK | South West (England) | 14.1 | 15.8 | 10.22 | 0 | 68.96 | 7.83 | 22.2 |
| UKL | Wales | 19.41 | 20.65 | 11.8 | 0 | 84.41 | 11.48 | 28.52 |
| UKM | Scotland | 15.5 | 23.07 | 21.7 | 0 | 92 | 6.35 | 33.45 |
| UKN | Northern Ireland | 15.47 | 16.84 | 9.45 | 0 | 80.45 | 9.89 | 22.24 |

# Supplementary Figures


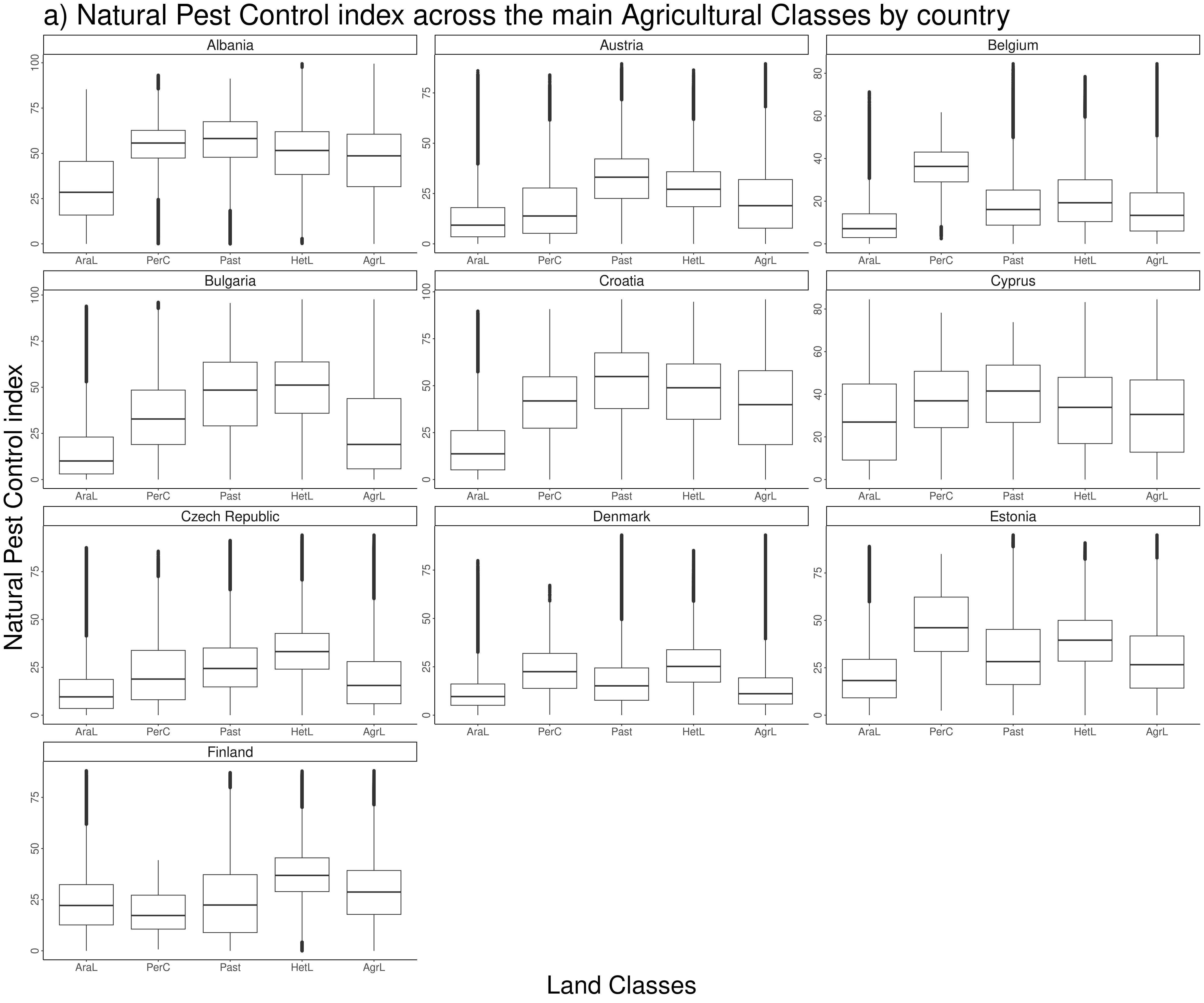

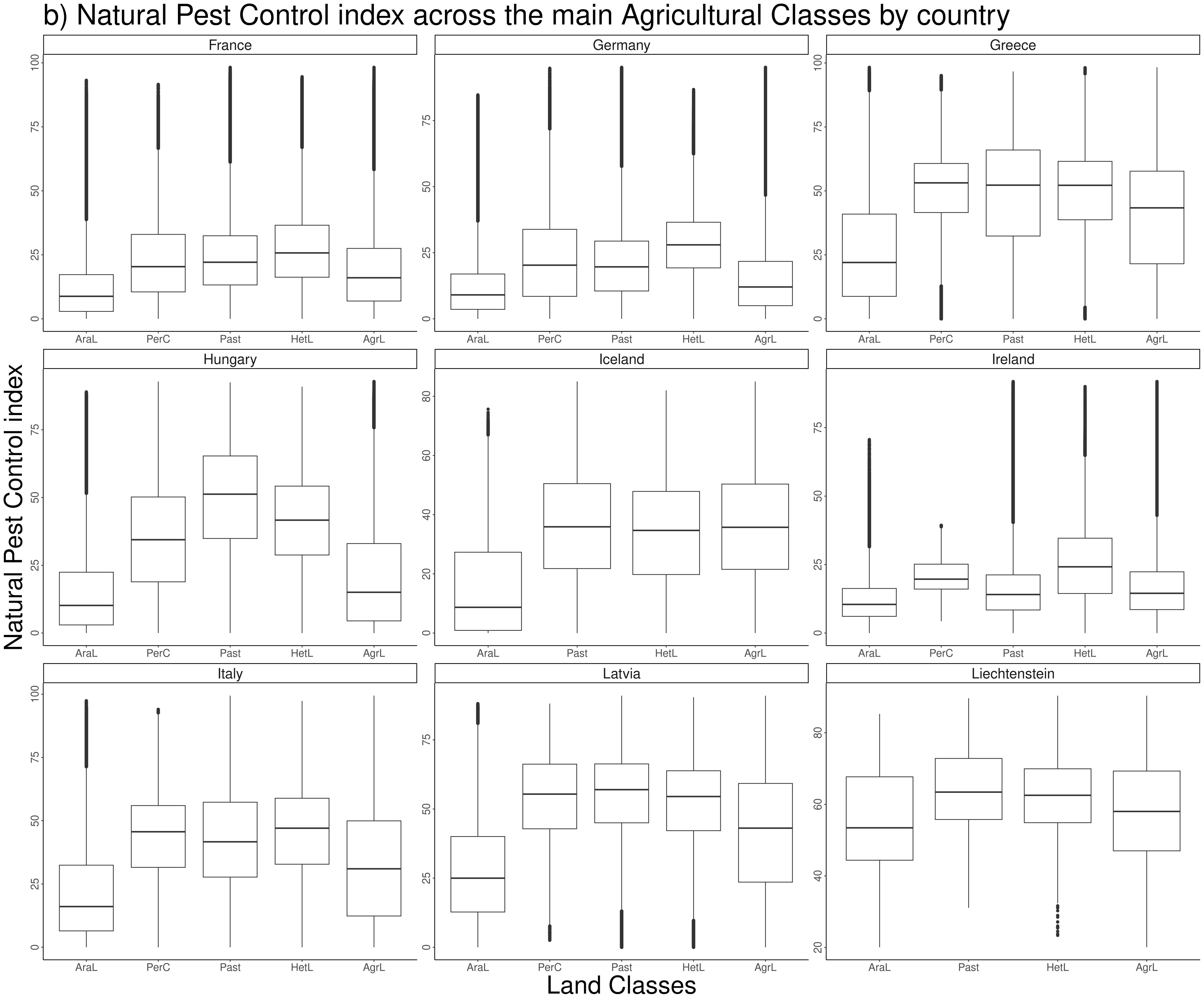

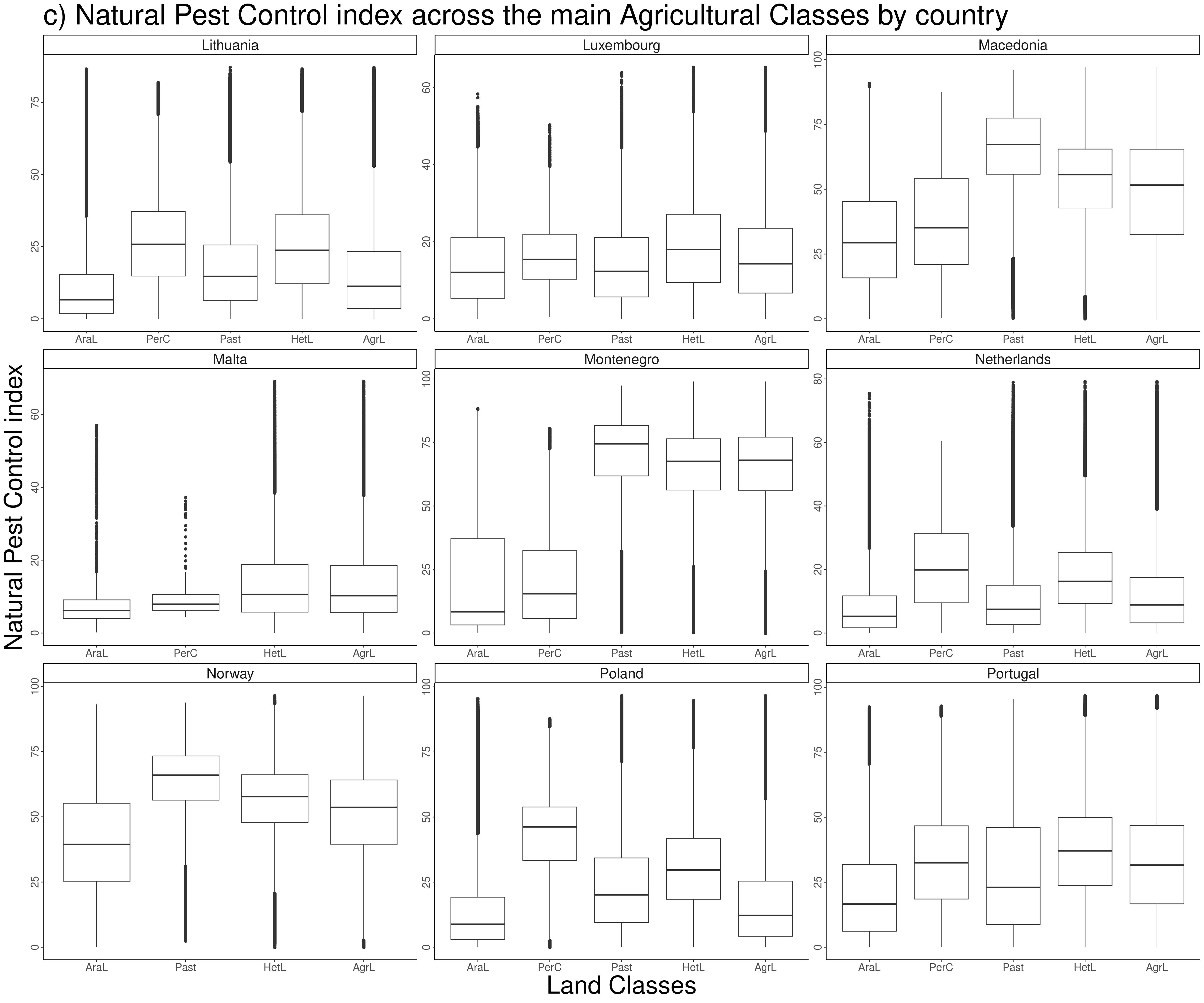

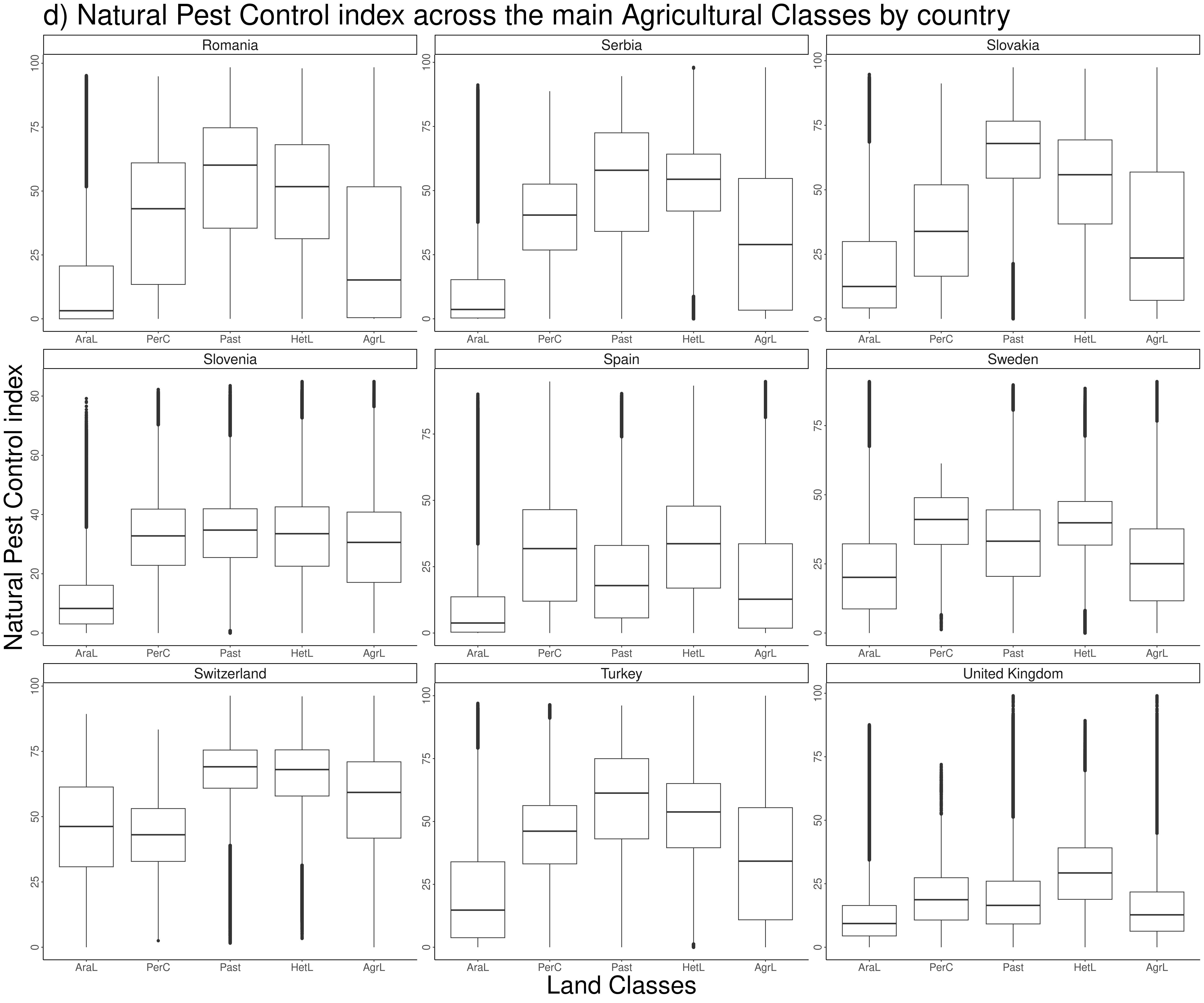


Supplementary Figure 1 a-d. Box plots illustrating the Natural Pest Control Index (NPCi) across main agricultural classes for each of the 36 countries, at the national scale, within pan-Europe. The agricultural classes include Arable Land (AraL), Permanent Crops (PerC), Pastures (Past), Heterogeneous Land (HetL), and Agricultural Land (AgrL). The NPCi values are shown on the y-axis, with the index ranging from 0 to 100. The box plots highlight the median, interquartile range, and potential outliers for each land class in each country, demonstrating the variability in natural pest control potential across different agricultural landscapes in Europe.

# Supplementary methods

# Supplementary method 1. Step 1 - Pseudo code for processing (combining) the WVM (Woody Vegetation Mask) and TCD (Tree Cover Density) layers

This section details the pseudo code for processing WVM (Woody Vegetation Mask) and TCD (Tree Cover Density) layers. This process generates a combined raster by tiling the input rasters, resampling TCD tiles, reclassifying values, merging tiles, and resolving overlaps using prioritization rules. The accompanying script (WVM_TCD_script.py), available at <https://jeodpp.jrc.ec.europa.eu/ftp/jrc-opendata/NPCI2018/?C=S;O=A>, emphasizes data preprocessing, reclassification, and final merging to ensure clarity and reproducibility.

1. **START**
2. **DEFINE paths to input files and directories:**

- WVM raster file (WVM_path)
- TCD raster file (TCD_path)
- Output directories for intermediate tiles and final results

1. **INITIALIZE necessary libraries and configurations (e.g., GDAL, NumPy)**
2. **DATA PREPROCESSING**
   1. TILE input rasters (WVM and TCD):

- Split WVM and TCD rasters into smaller tiles of specified height
- Save the tiles in designated directories
  1. RESAMPLE TCD tiles:
- Adjust the resolution of TCD tiles to match the WVM raster resolution
- Save the resampled tiles in the same directory

1. **RECLASSIFICATION AND COMBINATION**
2. RECLASSIFY TCD tiles:

- Set negative values to 0
- Reclassify specific values (e.g., 2 to 1) for alignment

1. RECLASSIFY WVM tiles:

- Set negative values to 0
- Set values greater than 1 to 0
- Reclassify specific values (e.g., 1 to 2) for alignment

1. COMBINE WVM and TCD tiles:

- Merge both tiles, prioritizing WVM values
- Resolve conflicts by taking the maximum value between corresponding pixels
- Reclassify merged values to ensure the output contains only valid values (e.g., 2s and 0s)
- Save the combined tiles in the output directory

1. **MERGING AND OUTPUT**
2. MERGE combined tiles into a single raster:

- Use GDAL tools to merge all combined tiles
- Apply compression for efficient storage

1. SAVE the final combined raster (e.g. WVM_TCD.tif) for analysis:

- Store the output raster in the specified directory

1. **CLEANUP**
2. DELETE temporary files and directories to free up storage
3. VERIFY the integrity of the output files
4. **OUTPUT**
5. Final combined raster file, ready for environmental and ecological analyses
6. **END**

# Supplementary method 2. Step 2 - Pseudo code for Morphological Spatial Pattern Analysis (MSPA) using GuidosToolbox (GTB)

This section details the pseudo code for conducting Morphological Spatial Pattern Analysis (MSPA) on the raster file *WVM_TCD.tif* (see step 1) using GTB. MSPA is a GIS-based methodology that analyses spatial patterns of binary data to identify key habitat structures, such as core areas, edges, and corridors. The process involves input preparation, binary conversion, MSPA parameter selection, and output generation.

1. **START**
2. **INPUT PREPARATION**
3. DEFINE input raster file:

- Combined raster file from WVM and TCD processing (WVM_TCD.tif)

1. b. VERIFY raster file compatibility with GTB software:

- Ensure the file is in a supported format (e.g., GeoTIFF)
- Check for correct binary values as required by MSPA analysis

1. IF raster values are not binary:

- Reclassify the raster using GIS tools (e.g., GDAL, QGIS, or GTB):
  - Set all missing data values to 0
  - Set all non-target values to 1
  - Set all target values to 2

1. **MSPA ANALYSIS USING GTB**
2. OPEN GTB and LOAD the binary raster file (`WVM_TCD_2018_binary.tif`)
3. Select the Quick Access/Pattern module
4. CONFIGURE MSPA parameters:

- Define pixel connectivity (e.g., 4-connectivity or 8-connectivity)
- Specify edge width for identifying habitat edges
- Choose to show transition pixels (1)
- Switch distinction of internal and external features to ON (1)

1. RUN MSPA analysis:

- GTB processes the binary raster based on the defined parameters
- Outputs a multi-class raster identifying different spatial patterns (e.g., Core, Edge, Bridge, Loop.

1. **OUTPUT GENERATION**
2. REVIEW and SAVE the MSPA output raster:

- Save the resulting raster file in a user-defined directory
- Ensure the output includes a legend or classification key for interpretation (see the MSPA Guide: https://ies-ows.jrc.ec.europa.eu/gtb/GTB/MSPA_Guide.pdf)

1. EXPORT additional data products if needed: - Generate statistics or summaries (e.g., total area of Core habitat)

- Export vectorised versions of specific classes for further GIS analysis

1. **POST-PROCESSING AND VERIFICATION**
2. VERIFY the integrity of the MSPA output file:

- Check for data completeness and alignment with the input raster

1. VISUALIZE results using GIS software (e.g., QGIS, ArcGIS):

- Overlay MSPA results with other spatial layers to interpret habitat patterns

1. **OUTPUT**
2. Final MSPA raster file, identifying spatial patterns for habitat structures
3. Summary statistics or vectorized layers (if required)
4. **END**

# Supplementary method 3. Step 3 - Pseudo code for processing the Semi-Natural Habitat (SNH) layer

This section details the pseudo code designed for processing the Semi-Natural Habitat (SNH) layer. The SNH layer is a critical component, providing a spatially explicit representation of habitats that support biodiversity, specifically by facilitating natural enemies. The pseudo code outlines the core processes involved in SNH layer generation, which include data preprocessing, habitat classification, spatial analysis, and final output preparation. The accompanying script (SNH_script.py) is available at <https://jeodpp.jrc.ec.europa.eu/ftp/jrc-opendata/NPCI2018/?C=S;O=A>.

1. **START**
2. **DEFINE paths to input files and directories:**

- MSPA raster file (MSPA_tif)
- Extensive grasslands raster file (extGL_tif)
- CORINE land cover raster file (CORINE_tif)
- Output directories for intermediate and final results

1. **INITIALIZE necessary libraries and configurations (e.g., GDAL, NumPy)**
2. **DATA PREPROCESSING**
3. SPLIT large rasters into smaller tiles:

- Divide MSPA_tif and extGL_tif into tiles of specified dimensions
- Save tiles in temporary directories

1. RECLASSIFY MSPA tiles:

- Categorize raster cells into Core, Edge, and Linear based on their values
- Save reclassified MSPA tiles

1. COMBINE reclassified MSPA tiles with extGL_tif tiles:

- Overlay MSPA and extensive grassland tiles
- Add values from extGL tiles to MSPA tiles while respecting rules (e.g., only where MSPA values allow)
- Save the resulting Semi-Natural Habitat (SNH) tiles

1. **SPATIAL ANALYSIS**
2. CREATE a binary CORINE raster:

Reclassify CORINE_tif to retain only specific land cover classes

Save the binary raster

1. MASK SNH tiles using the binary CORINE raster:

Apply the binary mask to SNH tiles

Remove regions that do not match the desired CORINE classes

Save the masked SNH tiles

1. **MERGING AND OUTPUT**
2. MERGE processed SNH tiles into a single raster:

Combine individual tiles into one unified raster file

1. SAVE the final SNH layer for analysis:

Ensure all files are compressed and stored in the output directory

1. **CLEANUP**
2. DELETE temporary files and directories to free up storage
3. VERIFY the integrity of the output files

# Supplementary method 4. Step 4 - Pseudo code for processing the Natural Pest Control Index (NPCi) layer

This section details the pseudo code designed for the calculation of the Natural Pest Control index (NPCi). The NPCi serves as a spatially explicit indicator that quantifies the potential of agricultural landscapes to support beneficial natural enemies, thereby enhancing natural pest control services. The pseudo code provided outlines the core processes involved in NPCi generation, including data pre-processing, habitat classification, spatial analysis, and index computation. The script (NPCi_script.py), available at <https://jeodpp.jrc.ec.europa.eu/ftp/jrc-opendata/NPCI2018/?C=S;O=A>, is accompanied by a self-explanatory description, ensuring clarity and reproducibility.

1. **Prepare the input files**

The first step involves preparing the necessary input files required for the calculation of the NPCi. These files should be placed in the same directory for organizational purposes, for example, *.../Data/Data_Input*. Below is a list of the required files, with a brief description of each:

- **Tree Cover Density (raster, 10 m resolution)**: Provides information on the density of tree cover across the landscape.
- **Woody Vegetation Mask (raster, 5 m resolution)**: Identifies areas covered by woody vegetation, such as shrubs and small trees.
- **Grasslands (raster, 5 m resolution)**: Maps the extent of grassland areas, important habitats for natural enemies.
- **CORINE Land Cover 2018 (raster, 100 m resolution)**: Offers a comprehensive classification of land cover types across Europe, used for habitat classification.
- **Nitrogen from CAPRI (raster, 1000 m resolution)**: Represents nitrogen levels in the landscape, which can impact vegetation and pest control dynamics.

For convenience, we provide pre-merged files available at [10.2905/963ed44c-b38f-4e9a-94db-990d5d0c93c8](https://doi.org/10.2905/963ed44c-b38f-4e9a-94db-990d5d0c93c8). However, if users need to work with different input files (e.g., from another year or geographical region), they will need to compile these into a single raster file. It is recommended that all input and output files share a common directory structure to maintain consistency and facilitate subsequent analysis.

1. **START**

DEFINE paths to input files and directories:

- MSPA raster file (MSPA_tif)
- Extensive grasslands raster file (extGL_tif)
- CORINE land cover raster file (CORINE_tif)
- Output directories for intermediate and final results

1. **INITIALIZE necessary libraries and configurations (e.g., GDAL, NumPy)**
2. **DATA PREPROCESSING**
   1. CREATE raster tiles:

- Divide MSPA_tif and extGL_tif into smaller tiles for efficient processing
- Save the tiles in designated directories
  1. RECLASSIFY MSPA tiles:
- Classify raster cells into Core, Edge, and Linear categories based on their values
- Apply Euclidean distance transformations for enhanced accuracy
- Save reclassified tiles
  1. COMBINE reclassified MSPA tiles with extGL_tif tiles:
- Add values from extGL tiles to MSPA tiles, ensuring compatibility
- Save the resulting Semi-Natural Habitat (SNH) tiles

1. **SPATIAL ANALYSIS**
   1. CREATE a binary CORINE raster:

- Reclassify CORINE_tif to retain only specific land cover classes
- Save the binary raster
  1. MASK SNH tiles using the binary CORINE raster:
- Apply the mask to SNH tiles, removing areas that do not match the desired classes
- Save the masked SNH tiles

1. **NPCi COMPUTATION**
   1. RESAMPLE SNH tiles to a consistent resolution (e.g., 50m)
   2. APPLY focal statistics:

- Load a pre-defined kernel for spatial analysis
- Perform convolution over resampled SNH tiles using the kernel
- Remove buffer zones introduced during preprocessing
- Save the resulting NPCi tiles
  1. MERGE processed NPCi tiles into a single raster
  2. NORMALIZE NPCi values:
- Scale the values of the merged raster to a range of 0-100
- Save the normalized raster

1. **CLEANUP**
   1. DELETE temporary files and directories to free up storage
   2. VERIFY the integrity of output files
2. **OUTPUT**
   1. Final NPCi raster file, normalized and ready for analysis
3. **END**
